# Epithelial NAD^+^ depletion drives mitochondrial dysfunction and contributes to intestinal inflammation

**DOI:** 10.3389/fimmu.2023.1231700

**Published:** 2023-09-07

**Authors:** Elizabeth A. Novak, Erin C. Crawford, Heather L. Mentrup, Brian D. Griffith, David M. Fletcher, Meredith R. Flanagan, Corinne Schneider, Brian Firek, Matthew B. Rogers, Michael J. Morowitz, Jon D. Piganelli, Qian Wang, Kevin P. Mollen

**Affiliations:** ^1^ Department of Surgery, University of Pittsburgh School of Medicine, Pittsburgh, PA, United States; ^2^ Division of Pediatric General and Thoracic Surgery, UPMC Children’s Hospital of Pittsburgh, University of Pittsburgh Medical Center, Pittsburgh, PA, United States; ^3^ Division of Gastroenterology, UPMC Children’s Hospital of Pittsburgh, University of Pittsburgh Medical Center, Pittsburgh, PA, United States; ^4^ Department of Surgery, University of Michigan School of Medicine, Ann Arbor, MI, United States; ^5^ University of Pittsburgh School of Medicine, Pittsburgh, PA, United States; ^6^ Department of Pathology, UPMC Children’s Hospital of Pittsburgh, University of Pittsburgh Medical Center, Pittsburgh, PA, United States

**Keywords:** colitis, PGC1α, nicotinamide riboside, poly(ADP) riboside polymers, nicotinamide adenine dinucleotide

## Abstract

**Introduction:**

We have previously demonstrated that a pathologic downregulation of peroxisome proliferator-activated receptor–gamma coactivator 1-alpha (PGC1α) within the intestinal epithelium contributes to the pathogenesis of inflammatory bowel disease (IBD). However, the mechanism underlying downregulation of PGC1α expression and activity during IBD is not yet clear.

**Methods:**

Mice (male; C57Bl/6, *Villincre*/+;*Pgc1afl/fl* mice, and *Pgc1afl/fl*) were subjected to experimental colitis and treated with nicotinamide riboside. Western blot, high-resolution respirometry, nicotinamide adenine dinucleotide (NAD+) quantification, and immunoprecipitation were used to in this study.

**Results:**

We demonstrate a significant depletion in the NAD+ levels within the intestinal epithelium of mice undergoing experimental colitis, as well as humans with ulcerative colitis. While we found no decrease in the levels of NAD+-synthesizing enzymes within the intestinal epithelium of mice undergoing experimental colitis, we did find an increase in the mRNA level, as well as the enzymatic activity, of the NAD+-consuming enzyme poly(ADP-ribose) polymerase-1 (PARP1). Treatment of mice undergoing experimental colitis with an NAD+ precursor reduced the severity of colitis, restored mitochondrial function, and increased active PGC1α levels; however, NAD+ repletion did not benefit transgenic mice that lack PGC1α within the intestinal epithelium, suggesting that the therapeutic effects require an intact PGC1α axis.

**Discussion:**

Our results emphasize the importance of PGC1α expression to both mitochondrial health and homeostasis within the intestinal epithelium and suggest a novel therapeutic approach for disease management. These findings also provide a mechanistic basis for clinical trials of nicotinamide riboside in IBD patients.

## Introduction

Inflammatory bowel disease (IBD), including Crohn’s disease (CD) and ulcerative colitis (UC), comprises a spectrum of chronic, relapsing, idiopathic inflammatory conditions of the gastrointestinal tract. It is estimated that 3.1 million Americans suffer from IBD, with the incidence and prevalence of disease increasing globally (1). Although new therapeutic alternatives have substantially transformed our approach to treating IBD in recent decades, 70–80% of CD patients and 20–30% of UC patients still progress to advanced, complicated disease requiring surgical intervention (2). Although the pathophysiology of IBD is complex and multifactorial, there is a growing body of evidence that implicates mitochondrial dysfunction as a key intermediate in disease development and progression (3). Mitochondrial dysfunction can alter cellular signaling pathways through metabolites and ROS, disrupt the intimate physical connections between the mitochondria and other cellular organelles, and negatively impact the bioenergetics of the cell (3). A wide range of clinical conditions are associated with mitochondrial dysfunction, including cancer, diabetes, cardiomyopathy, neurogenerative disorders, and muscular disorders (3, 4). However, the role mitochondrial dysfunction plays in IBD is still not fully understood.

Our previously published work demonstrated a failure in mitochondrial biogenesis and bioenergetics during the pathogenesis of colitis (5). Mitochondrial biogenesis, the process by which new mitochondria are generated and repaired, provides the cell with an adequate pool of healthy mitochondria and is critical to cellular homeostasis (3). Mitochondrial biogenesis involves precise communication between the nucleus and mitochondria (6–8). PGC1α (peroxisome proliferator-activated receptor gamma coactivator 1-alpha) is a central regulator of mitochondrial biogenesis, driving the activity of other transcription factors, specifically TFAM (mitochondrial transcription factor A), to promote the transcription and replication of the mitochondrial genome (9). We previously demonstrated a pathologic downregulation of PGC1α within the intestinal epithelium in humans with UC and mice undergoing experimental colitis (5). PGC1α expression has also been shown to be strongly associated with complicated forms of CD (10). Additionally, colonic mitochondriopathy was observed in newly diagnosed UC patients, with significant suppression of all 13 mitochondrial DNA-encoded genes that regulate ATP production (complex I, III, IV, and V), as well as PGC1α (11). Alterations in PGC1α gene expression are now recognized to be present at diagnosis and the extent of downregulation correlates with disease severity (11). Yet, the mechanism underlying the downregulation of PGC1α expression and activity during IBD is not yet clear.

PGC1α expression and activity are regulated at both the transcriptional and posttranslational level, with reversible acetylation being a key manner in which PGC1α protein is regulated (12, 13). PGC1α possesses 13 lysine residues, and the acetylation of these sites has been shown to negatively correlate with the activity of PGC1α (13). Further, the acetylation status of PGC1α is an important indicator of the functionality of mitochondrial biogenesis (14). We previously demonstrated that PGC1α expression is downregulated within the intestinal epithelium of mice undergoing experimental colitis and in humans with UC. We further demonstrated an increase in the levels of acetylated (inactive) PGC1α and mitochondrial dysfunction within the intestinal epithelium of mice subjected to experimental colitis (5). Since PGC1α protein is primarily activated through deacetylation via the nicotinamide adenosine dinucleotide (NAD^+^)-dependent deacetylase, sirtuin 1 (SIRT1) (12, 13), we speculated that a defect in SIRT1 may be responsible for the decreased PGC1α activity during intestinal inflammation (5). Building on our previous study, we show here that both the transcript and protein levels of SIRT1 are decreased in murine models of chemically induced and infectious colitis. We also demonstrate a significant decrease in the NAD^+^ levels within the intestinal epithelium during colitis. Furthermore, treating mice undergoing experimental colitis with an NAD^+^ precursor, nicotinamide riboside (NR), decreased the severity of colitis, restored mitochondrial function, and decreased the levels of acetylated (inactive) PGC1α. Additionally, NR treatment did not benefit mice with a conditional knockout of PGC1α within the intestinal epithelium undergoing experimental colitis, suggesting that the effects of NR require an intact PGC1α axis. Based on our following studies, we now hypothesize that significantly decreased NAD^+^ levels within the intestinal epithelium during colitis renders SIRT1 unable to activate PGC1α, resulting in decreased mitochondrial biogenesis and subsequent mitochondrial dysfunction. Thus, future therapeutic approaches targeting the activity of PGC1α, and in turn mitochondrial health, may complement the treatment for IBD and improve outcomes in patients.

## Materials and methods

### Mouse strains and husbandry

WT C57Bl/6J male mice were purchased from The Jackson Laboratory (cat. #: 000664; Bar Harbor, ME, USA). Our lab previously generated the *Villin^cre/+^*;*Pgc1α^fl/fl^* mice (*Pgc1α^ΔIEC^*; transgenic mice with *Pgc1α* selectively deleted from the intestinal epithelium) and *Pgc1α^fl/fl^* mice (WT littermates), both on a C57BL/6J background (5). The strains were bred and maintained in the rodent barrier corridor at the University of Pittsburgh Medical Center (UPMC) Children’s Hospital of Pittsburgh Rangos Research Building, an Association for the Assessment and Accreditation of Laboratory Animal Care International-accredited facility. Mice used in the infectious colitis model were housed in the rodent biosafety level 2 corridor of the animal vivarium. All mice were housed (2–4 mice/cage) in individually ventilated micro-isolator cages in a thermoneutral environment with a 12-hour light/dark cycle. Mice were provided food and water ad libitum throughout the experiment. All manipulations (e.g., observation, weighing, treatments) were performed at the same time every day to minimize interruptions to the natural murine circadian rhythm.

WT mice purchased from The Jackson Laboratory were randomized upon arrival using the GraphPad online random number generator (https://www.graphpad.com/quickcalcs/randomize1/) and then allowed to acclimate to their environment for 5–7 days prior to the start of experiments. *Pgc1α^ΔIEC^* and *Pgc1α^fl/fl^* mice were randomized across genotype and litter using the above online random number generator. Once randomized, all cages were changed every other day for at least one week to allow the gut microbiota to normalize prior to the start of experimentation.

### Dextran sulfate sodium-induced acute colitis

Acute colitis was induced in WT C57BL/6j, *Pgc1α^ΔIEC^*, and *Pgc1α^fl/fl^* male mice (7–8 weeks old, 20–25 grams) by the addition of 2% DSS (molecular weight, 35–45 kilodaltons; Thermo-Fisher Scientific, Waltham, MA, USA) to the drinking water of mice for 7 days ad libitum as described previously (5, 15, 16). Clinical signs of colitis were recorded daily, and the disease activity index (DAI) score, which included weight loss (0: <1%, 1: 1–5%, 2: 5–10%, 3: 10–15%, 4: >15%), stool consistency (0: formed and hard, 1: formed and soft, 2: loose stools, 3: mild diarrhea, 4: gross diarrhea), and blood in the stool (0: no blood, 2: positive fecal occult, 4: gross blood in stool), was calculated daily. Weight loss was calculated as the percent weight change from the original weight before initiation of the experiment. Mice were euthanized after 7 days of DSS treatment by CO_2_ asphyxiation. Colon lengths were measured, and then the colon was either preserved for histology or colonic mucosal scrapings were removed and snap-frozen for RNA and protein isolation and stored at −80°C or harvested and processed immediately for metabolic assays.

To determine the effects of NAD^+^ supplementation on the development and progression of experimental colitis, mice undergoing DSS colitis were treated with a NAD^+^ precursor, nicotinamide riboside (NR; 500 mg/kg/day; Chromadex, Los Angeles, CA, USA), or vehicle (sterilized water from the animal facility; same volume per kg as NR dosage). For NR treatment studies, the mice were divided into four groups: 1) Vehicle Control group (control mice treated with the vehicle), 2) NR Control group (control mice treated with NR), 3) DSS+Vehicle group (mice subjected to DSS and treated with the vehicle), and 4) DSS+NR group (mice subjected to DSS and treated with NR). Oral treatment of NR or vehicle started when DSS started (Day 0) and occurred every day, with the last treatment on day 6. Mice were euthanized on Day 7, 24 hours after the last treatment. DAI scores and weight loss were calculated as described for the DSS-induced colitis model. Colon lengths were measured, and then the colon was either preserved for histology or the colonic mucosal tissues were removed and snap-frozen for RNA and protein isolation or harvested and used immediately for metabolic assays.

### Growth and quantification of *Citrobacter rodentium*

A frozen glycerol stock of kanamycin-resistant *C. rodentium* DBS100 (a kind gift from Dr. Vanessa Sperandio, University of Wisconsin, School of Medicine and Public Health, Madison, WI, USA) was streaked onto a Luria Agar (LA; Sigma-Aldrich, St. Louis, MO, USA) plate supplemented with kanamycin (100 µg/ml; Sigma-Aldrich) and incubated for 18 hours at 37°C. A single colony was then selected from the plate, inoculated into Luria Broth (LB; Sigma-Aldrich), and incubated for 18 hours in a 37°C shaker at 220 rpm. This overnight culture was then used to start a fresh culture in LB for growth curve analysis or mouse infections. The LA plate with single colonies was stored at 4°C for 10 days, after which it was discarded.

To determine the colony-forming units (CFUs) per milliliter (CFUs/ml), an overnight culture (grown for 18 hours) was inoculated 1:20 in fresh LB medium supplemented with kanamycin (100 µg/ml). The culture was incubated in a 37°C shaker at 220 rpm. At 30-minute time periods, the optical density at 600 nm (OD_600nm_) was measured with a Spectramax M2 Microplate Reader equipped with Softmax Pro Data Acquisition and Analysis Software (Molecular Devices, Sunnyvale, CA, USA). An aliquot of the culture was also serially diluted, plated on LA plates, and incubated overnight at 37°C. The colonies were then counted and recorded. The OD_600nm_ measurements were plotted against the CFUs/ml at each time point in order to obtain a growth curve. A standard curve equation, derived from the linear portion of the growth curve, was used to determine the CFUs/ml of the *C. rodentium* cultures used in the mouse studies.

### Infectious colitis model

On the day of infection (Day 0), an overnight culture of *C. rodentium* was inoculated 1:20 in fresh LB medium (Sigma-Aldrich) supplemented with kanamycin (100 µg/ml; Sigma-Aldrich). Once the cultures were in late exponential phase, the cultures were centrifuged at 3,000 × g for 10 minutes, and the pellets were resuspended in sterile Dulbecco’s PBS (dPBS; Thermo-Fisher Scientific). Mice were randomized and housed as described above. WT C57Bl/6J male mice (6 weeks old) were assigned to either a Sham-infected group or a *Citrobacter*-infected group. Sham-infected mice were gavaged 200 µl of 1× dPBS, and each *Citrobacter*-infected mouse was orally gavaged with *C. rodentium* (1 × 10^9^ CFUs in 200 µl of 1× dPBS). Clinical signs of colitis were recorded daily, and DAI scores, as described above, were calculated daily. Mice were euthanized 8 days after infection. Weight loss was calculated as the percent change from the original weight before initiation of the experiment. Stool was collected on Days 0, 3, 5, and 8 to determine the fecal bacterial burden. Mice were euthanized by CO_2_ asphyxiation on Day 8 post-infection. Colon lengths were measured, and then the colon was either preserved for histology or the colonic mucosal tissues were removed and snap-frozen for RNA and protein isolation or harvested and used immediately for metabolic assays.

Mice undergoing the infectious colitis model were also treated with NR (500 mg/kg/day) or vehicle (sterilized water from the animal facility; same volume per kg as NR dosage). For NR treatment studies, the mice were divided into four groups: 1) Sham+Vehicle control group (control mice sham-infected with 1× dPBS and treated with the vehicle), 2) Sham+NR control group (control mice sham-infected with 1× dPBS and treated with NR), 3) *Citrobacter*+Vehicle group (mice orally infected with *C. rodentium* and treated with the vehicle), and 4) *Citrobacter*+NR group (mice orally infected with *C. rodentium* and treated with NR). Oral treatment of NR or vehicle started 24 hours after infection (Day 1) and occurred every day, with the last treatment on Day 7. Mice were euthanized on Day 8, 24 hours after the last treatment. DAI scores and weight loss were calculated as described for the infectious colitis model. Colon lengths were measured, and then the colon was either preserved for histology or the colonic mucosal tissues were removed and snap-frozen for RNA and protein isolation or harvested and used immediately for metabolic assays.

### Determination of fecal bacterial burden

For quantification of *C. rodentium* in stool, fresh stool pellets were collected from each mouse. The stool was resuspended and homogenized in 1× sterile dPBS at a ratio of 0.1 g of stool per 1 ml of dPBS and then serially diluted in dPBS and plated on LA plates for CFU counting. The LA plates were incubated overnight at 37°C. The CFU stool burden of *C. rodentium* was determined by counting colonies from the triplicate plating of a serial dilution and normalizing the colonies to stool weight.

### RNA isolation, lithium chloride precipitation, and cDNA synthesis

Total RNA was isolated from murine colonic tissue scrapings and human intestinal biopsies using the RNeasy Kit (Qiagen, Germantown, MD, USA), according to the manufacturer’s instructions. Since DSS is co-extracted with nucleic acids when administered *in vivo* and the concentration of DSS extracted with murine tissue RNA is sufficient to inhibit downstream applications (17–19), we precipitated the isolated RNA from mouse tissue with LiCl (Sigma-Aldrich). Once isolated, 0.25 volumes of 8M LiCl was added to the RNA. The samples were incubated at −20°C for 30 minutes, centrifuged at 4°C at 15000 rpm, and the supernatant was discarded. The RNA pellet was resuspended in ultrapure water (Invitrogen, Carlsbad, CA, USA) with 0.25 volumes of 8 M LiCl and incubated at −20°C for 1 hour. Then the samples were centrifuged at 4°C at 15000 rpm, and the supernatant was discarded. The RNA pellet was resuspended in ultrapure water and precipitated overnight at −20°C with 0.1 volume of 3 M sodium acetate, pH 5.2, and 2 volumes of 100% ethanol. Samples were then centrifuged at 15000 rpm at 4°C for 30 minutes. The pellets were washed twice with 70% ethanol to remove residual salt, air-dried for 15 minutes, and finally resuspended in ultrapure, RNase-free water. After isolation, human RNA was further purified using the Dynabeads mRNA Purification Kit (Invitrogen), and then the enriched mRNA was used for cDNA synthesis.

The concentration and purity of each RNA sample were measured via spectrophotometry (ND-2000 spectrophotometer; NanoDrop Technologies, Inc., Wilmington, DE, USA). First-strand cDNA (0.5 µg of RNA) was prepared by using a QuantiTect Reverse Transcription Kit (Qiagen) according to the manufacturer’s instructions. cDNA was diluted to 0.5 ng/µl and stored at −20°C until further use.

### Real-time quantitative PCR and analysis

Gene expression was measured relative to the housekeeping gene 50s ribosomal subunit protein L15 (*Rplp0*). Primers used in this study are listed in **Table S1**. cDNA was amplified using a QuantStudio Real-time PCR System (Thermo-Fisher Scientific), with a final reaction volume of 10 µl containing 2.5 ng of cDNA, primers (500 nM final concentration), and 1× PowerUp SYBR Green Master mix (Thermo-Fisher Scientific). The amplification conditions for the quantitative PCR (qPCR) reactions were as follows: 1 cycle of initial denaturation at 95°C for 20 sec followed by 40 cycles of denaturation at 95°C for 1 s and annealing and extension at 60°C for 20 s. A melt curve was performed after each reaction protocol by incrementally and continuously increasing the temperature 0.1°C/cycle from 60–95°C. All reactions were performed in triplicate. Primer efficiencies were validated to be similar for all primers in comparison to *Rplp0*, which allowed the qPCR data to be analyzed by the comparative threshold (ΔΔ*C_t_*) method. The derived ΔΔ*C_t_* values were converted into fold-difference values.

### SDS-PAGE and immunoblot

Protein lysates were prepared from murine intestinal epithelial scrapings in 1× RIPA buffer (Boston Bio, Ashland, MA, USA). The protein concentration was determined using a bicinchoninic acid assay (BCA; Sigma-Aldrich). Protein lysates (10–40 µg/sample) were separated on 8% or 10% SDS gels by SDS-PAGE and transferred onto 0.45-µM polyvinylidene difluoride (PVDF; Millipore, Burlington, MA, USA) membranes. The membranes were blocked with 5% nonfat dry milk in 1× Tris-buffered saline with Tween 20 (TBST; 20 mM Tris, 150 mM NaCl, 0.1% Tween-20) and probed with a primary antibody at 4°C overnight. The membranes were then incubated with a horseradish peroxidase (HRP)-conjugated secondary antibody (Cell Signaling Technology, Danvers, MA, USA) for 1 hour, followed by incubation with a chemiluminescent HRP substrate (Thermo-Fisher Scientific). Protein bands were acquired with a Kodak X-Omat 2000 processor (Eastman Kodak Company, Rochester, NY, USA). The relative intensity of protein bands was quantified and analyzed using ImageJ (National Institutes of Health, Bethesda, MD, USA; http://rsb.info.nih.gov/jj/). Relative band intensity was calculated as the ratio of the total target protein to β-actin.

### Histology

Murine colonic tissue was fixed in 4% paraformaldehyde (Electron Microscopy Services, Hatfield, PA, USA) overnight. The next day the tissues were washed in 1× PBS, dehydrated in 70% ethanol (Sigma-Aldrich), and then embedded in paraffin blocks. Tissue sections were cut at 5–10-µm thickness on a HM325 rotary microtome (Thermo-Fisher Scientific), deparaffinized, and rehydrated through a gradient of xylene and ethanol baths. The tissue sections were then stained with hematoxylin and eosin (H&E; Sigma-Aldrich), dehydrated, and mounted using toluene (Thermo-Fisher Scientific) for examination via light microscopy. Tissues were evaluated in a blinded manner by a pathologist for signs of disease, as previously described (5, 15, 16, 20). Histology scoring was as follows: 0, no signs of inflammation; 1, very low levels of inflammation; 2, low levels of leukocyte infiltration; 3, high levels of leukocyte infiltration, high vascular density, and thickening of colon wall; 4, transmural infiltration, loss of goblet cells, high vascular density, and thickening of the colon wall (20). Images were acquired with a light microscope (Leica DFC9000 GT, Leica Microsystems, Buffalo Grove, IL, USA).

### Assessment of acetylated PGC1α levels

Protein lysates were prepared by homogenizing approximately 50 mg of colonic mucosal scrapings from control mice and mice subjected to experimental colitis in 250 µl of Tissue-Protein Extraction Reagent (T-PER; Thermo-Fisher Scientific) with protease and phosphatase inhibitors. The protein lysates were centrifuged for 15 minutes at 15000 rpm at 4°C, and the supernatant was collected. The protein concentration was determined via a BCA assay (Thermo-Fisher Scientific). The lysates were resuspended at a concentration of 1 mg/ml and precleared by incubating with 50 µl of Agarose A Sepharose beads (Sigma-Aldrich) at 4°C for 3 hours. The lysate-bead complexes were centrifuged at 3500 rpm for 3.5 minutes, and the supernatant was collected. An aliquot (10 µg) of each precleared protein lysate was set aside as the input for the immunoprecipitation. Then 500 µg of the precleared protein lysate was added to a new tube with either 2 µg of PGC1α antibody (cat. #: ST1204, Sigma-Aldrich) or 2 µg rabbit IgG isotope control antibody (cat. #: 2729S, Cell Signaling Technology) overnight at 4°C on a rotator. The next day the antibody-protein complexes were incubated with 50 µl of Agarose A Sepharose beads at 4°C for 3 hours. The protein-bead complexes were centrifuged at 3500 rpm for 3.5 minutes, washed three times with T-PER lysis buffer with protease and phosphatase inhibitors, and then washed three times with 1× PBS. Finally, the immunoprecipitated protein was eluted in 2× Laemmli buffer at 95°C for 5 minutes, and the eluted protein was separated from the beads by centrifuging at 3500 rpm for 3.5 minutes. The immunoprecipitated protein was resolved via SDS-PAGE, transferred onto a PVDF membrane, and the levels of acetylated PGC1α were assessed by incubating the membrane with an anti-acetylated lysine antibody [1:1000 (cat #: 9441S, Cell Signaling Technology); diluted in 5% BSA] and anti-PGC1α antibody [1:500 dilution (cat. #: ab191838, Abcam); diluted in 5% milk]. The levels of acetylated PGC1α were calculated as the ratio of acetylated-lysine PGC1α to total PGC1α protein using ImageJ to measure the band intensities.

### SIRT1 activity assay

SIRT1 was immunoprecipitated from the colonic mucosal scrapings of control and DSS mice and subjected to a commercially available SIRT1 Activity Assay Kit (Sigma-Aldrich). Colonic mucosal scrapings were homogenized in cell lysis buffer (20 mM Tris-HCl, pH 8.0, 137 mM NaCl, 10% glycerol, 1% NP-40, and 2 mM EDTA). SIRT1 was immunoprecipitated from the colonic mucosal scrapings following the protocol provided by the manufacturer, with a few modifications. The lysates were resuspended at a concentration of 1 mg/ml and precleared by incubating with 50 µl of Agarose A Sepharose beads (Sigma-Aldrich) at 4°C for 3 hours. The lysate-bead complexes were centrifuged at 3500 rpm for 3.5 minutes, and the supernatant was collected. Then 500 µg of the precleared protein lysate was added to a new tube with either SIRT1 antibody (2 μg; cat. #: 8469, Cell Signaling Technology) or rabbit IgG isotope control antibody (2 μg; cat. #:2729S, Cell Signaling Technology) overnight at 4°C on a rotator. The next day the antibody-protein complexes were incubated with 50 µl of Agarose A Sepharose beads at 4°C for 3 hours. The protein-bead complexes were centrifuged at 3500 rpm for 3.5 minutes, washed three times with cell lysis buffer, one time with 1× PBS, and then one time with 1× SIRT1 Assay Buffer. The bead-antibody-protein complexes were suspended in 1× SIRT1 Assay Buffer and used directly in the assay. The assay was set up following the manufacturer’s instructions. Stop solution was added to the reaction when the velocity was stable, and the fluorescence intensity was measured (excitation = 340 nm; emission = 450 nm).

### High-resolution respirometry

The mitochondrial respiration from freshly harvested colonic mucosal scrapings was measured using an Oxygraph-2K (O2k, Oroboros Instruments, Innsbruck, Austria) as previously described (11). Each chamber was air-calibrated in Mir05 respiration medium (0.5 mM EGTA, 3 mM MgCl2, 60 mM potassium lactobionic acid, 20 mM taurine, 10 mM KH_2_PO_4_, 20 mM HEPES, 110 mM sucrose, 0.1% fatty acid-free BSA) before each experiment. All experiments were performed at 37°C. Colonic mucosal scrapings from control mice and experimental mice were homogenized in Mir05 respiration medium, briefly centrifuged to pellet any debris, and then approximately 300–400 µg of the homogenate was added into the respiration chamber with 2 ml of Mir05 medium. Once baseline oxygen levels in each chamber became stable, cytochrome *c* (10 µM) was added to assess mitochondrial integrity, and if the samples showed minimal damage, then malate (2 mM), pyruvate (5 mM), ADP (5 mM), and glutamate (10 mM) were added in rapid succession to stimulate respiration through Complex I. Upon reaching steady state, succinate (10 mM) was added to assess the combined activity of Complexes I + II. Next, rotenone (1 mM) was added to inhibit Complex I activity, and additional succinate was added to assess the maximal activity of Complex II. Carbonyl cyanide p-trifluoromethoxyphenylhydrazone (0.5 µM) was then added to uncouple the mitochondrial membrane and induce maximal respiration. Respiration rates were normalized to the amount of protein added for each sample. Complex I respiration was defined as malate/pyruvate/glutamate-driven oxygen consumption in the presence of ADP, whereas Complex II respiration was defined as succinate-driven oxygen consumption. Average rates of oxygen consumption [(pmol/(s×ml)/µg protein] ± SD were graphed.

### Determination of NAD^+^/NADH levels

NAD^+^/NADH levels in the colonic epithelium were assessed by using a NAD^+^/NADH Kit (Abcam) following the manufacturer’s instructions. Intestinal epithelial scrapings were homogenized in NAD^+^ Extraction Buffer (provided in the kit) supplemented with protease and phosphatase inhibitors. NAD^+^ levels were normalized to the protein concentration of the protein lysates prior to NAD^+^/NADH extraction.

### DNA extraction for 16S analysis

Stool pellets were collected from mice (n=8/group) in the Vehicle Control, NR Control, DSS+Vehicle, and DSS+NR groups on Days 0 and 7 of the acute DSS-induced model. DNA was extracted from fecal stool samples using the DNeasy 96 PowerSoil Pro QIAcube HT DNA Extraction Kit (Qiagen) according to the manufacturer’s instructions with one modification. Prior to the homogenization step, samples were heated at 65°C for 10 minutes in a bead bath. The DNA concentration of each sample was then quantified using the Invitrogen Quant-iT dsDNA Assay High Sensitivity Kit (Thermo-Fisher Scientific).

### 16S amplicon PCR and sequencing

All 16S rRNA Illumina-tagged PCR reactions were performed on fecal DNA following a protocol previously published by the Earth Microbiome Project. PCR products were resolved on a 2% agarose gel, pooled, and gel purified using the Qiagen Gel Extraction kit (Qiagen). Prior to sequencing, the purified pooled samples underwent quality assessment using an Agilent 2100 BioAnalyzer and Agilent DNA High Sensitivity DNA Kit (Agilent Technologies, Santa Clara, CA, USA). The purified samples were stored at −20°C until sequenced by Wright Labs (Huntingdon, PA, USA) using Illumina MiSeq v2 chemistry with paired-end reads of 250 bp.

### Analysis of 16S sequences

In order to increase the size and improve the quality of the 16S amplicons, forward and reverse reads were stitched together using PEAR, demultiplexed using QIIME (version 1.9), and quality-filtered using UPARSE (version 8.0). UPARSE was then employed to dereplicate and cluster the reads into operational taxonomic units (OTUs) at an identify threshold of 0.97 and remove chimeric reads. An OTU table was generated, and taxonomic assignments were completed via QIIME using the UCLUST method. α diversity, β-diversity distances, and principal coordinate analyses (PCoA) were calculated using QIIME. Microbiome analyses were performed using the Vegan and Phyloseq libraries in R. Generation of an OTU table, phylogenetic tree, and taxonomic predictions were done using the QIIME2 package. Differences in β-diversity were tested using the ADONIS2 test, and group homogeneity was tested using the PERMDISP test.

### Graphical illustrations

All graphical illustrations were created using Biorender.com.

### Statistical analysis

Data were statistically analyzed using GraphPad Prism 9.0 (San Diego, CA, USA). The data were analyzed by either a two-tailed unpaired *t* test for two groups, one-way analysis of variance (ANOVA) for multiple groups with one independent variable followed by Tukey’s *post-hoc* comparison, or a two-way ANOVA for multiple groups with two independent variables followed by Tukey’s *post-hoc* comparison. Statistical significance was accepted at *p* < 0.05 between groups in all experiments, and *p*-values were provided as * *p* < 0.05, ** *p* < 0.005, and *** *p* < 0.0005. Results are expressed as the mean ± SD, unless noted otherwise.

### Human tissue procurement

Human studies were approved by the Institutional Review Board of the University of Pittsburgh and UPMC’s Children’s Hospital of Pittsburgh. Human intestinal tissue biopsy specimens were acquired from patients undergoing a routine colonoscopy procedure or surgery at UPMC Children’s Hospital of Pittsburgh for either IBD or another diagnosis, which, if uninflamed, served as age-matched, non-diseased controls. Patients 18 years and younger were enrolled with 2-parent written informed consent. No samples or patient identifiable information were collected until a valid informed consent was signed by both parents (unless one parent: died, or was unknown, incompetent, not reasonably available, or had sole legal custody of the child). Exclusion criteria included the following: (1) lack of parental consent, (2) congenital anomalies, and (3) pregnant females. All patient samples were de-identified and handled in a blinded manner with the coding system only being known to the research coordinator. Samples were processed and analyzed without knowledge of the patient’s private information. Final pathology reports were used to group patient samples according to the IBD subset.

### Study approval

All animal studies were approved by the Institutional Animal Care and Use Committee (IACUC) of the University of Pittsburgh and conducted in accordance with the guidelines set forth by the Guide for the Care and Use of Laboratory Animals. Human studies were approved by the IRB of the University of Pittsburgh and UPMC’s Children’s Hospital of Pittsburgh.

## Results

### NAD^+^ levels are decreased during the pathogenesis of experimental colitis

**Figure 1:**
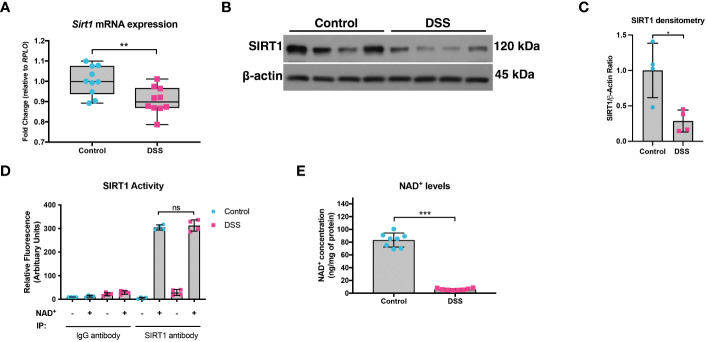
SIRT1 mRNA and protein levels were decreased during experimental colitis. Acute colitis was induced by giving mice 2% DSS in their water for 7 days. **(A)** *Sirt1* mRNA levels (n=10/group) and **(B, C)** protein levels (n=4/group) were decreased in mice subjected to DSS as compared to control mice. **(D)** The deacetylase activity of SIRT1 was assessed in SIRT1 protein immunoprecipitated from the colonic mucosal scrapings of control and DSS mice (n=4/group). **(E)** NAD^+^ levels within the colonic mucosa of control and DSS mice were evaluated using a NAD^+^/NADH Quantification Kit (n=8/group). Results are shown as the mean ± SD. The data are representative of at least two independent experiments. Significance differences between groups (as determined by an unpaired two-tailed unpaired *t* test) are indicated on the graphs. * *p* < 0.05, ** *p* < 0.005, and *** *p* < 0.0005; ns, not significant.

We previously demonstrated that not only is PGC1α expression decreased in the intestinal epithelium of mice undergoing experimental colitis and in humans with UC, but that there is also an increase in the levels of acetylated (inactive) PGC1α and mitochondrial dysfunction in mice subjected to DSS colitis (5). Since PGC1α protein is primarily activated through deacetylation via the deacetylase SIRT1 (13, 21), we hypothesized that there may be a defect in the activity of SIRT1. Thus, we subjected mice to a model of acute DSS colitis and assessed the expression of SIRT1. We found a significant decrease in both the mRNA and protein levels of SIRT1 in mice undergoing experimental colitis (**Figures 1A-C**), which is consistent with published reports (22–25). We also evaluated the deacetylase activity of SIRT1 and found no difference in the deacetylase activity of SIRT1 immunoprecipitated from the intestinal epithelium of DSS mice when supplied with ample NAD^+^ as compared to control mice (**Figure 1D**). SIRT1 protein levels and activity are known to be profoundly influenced by cellular NAD^+^ levels (26, 27), as SIRT1 utilizes NAD^+^ as a cofactor for its deacetylase activity (28). As such, we assessed the NAD^+^ levels within the intestinal epithelium of mice subjected to DSS and indeed found significantly lower levels of NAD^+^ in the intestinal epithelium of mice subjected to experimental colitis as compared to control mice (**Figure 1E**). This suggests that decreased NAD^+^ levels within the intestinal epithelium during inflammation may be responsible for decreased SIRT1 activity and increased acetylation levels of PGC1α.

### PARP1 expression and activity is increased during experimental colitis

**Figure 2:**
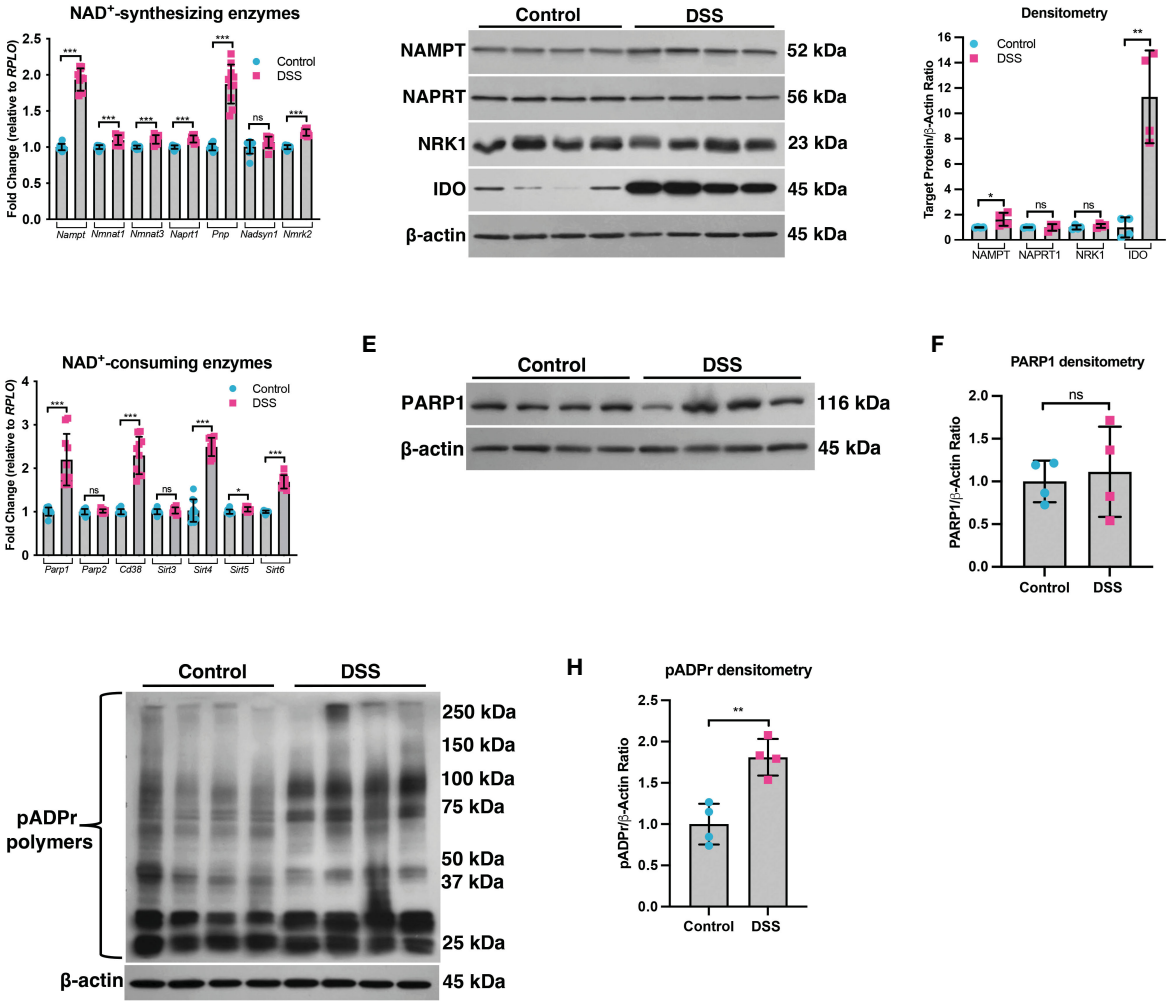
The expression of NAD^+^-synthesizing and NAD^+^-consuming enzymes in the intestinal epithelium during experimental colitis. Acute colitis was induced by giving mice 2% DSS in their water for 7 days. **(A)** The mRNA (n=10/group) and **(B, C)** protein levels (n=4/group) of several NAD^+^-synthesizing enzymes were assessed. **(D)** The mRNA expression (n=10/group) of NAD^+^-consuming enzymes was assessed. **(E, F)** The protein levels (n=4/group) of the NAD^+^-consuming protein, PARP1, within the intestinal epithelium of DSS mice as compared to the control mice were determined. **(G, H)** Western blot analysis of poly(ADP)-ribose (pADPr)-modified proteins within the intestinal epithelium of mice subjected to DSS as compared to control mice (n=4/group). Results are shown as the mean ± SD. The data are representative of at least two independent experiments. Significance differences between groups (as determined by an unpaired two-tailed *t* test) are indicated on the graphs. * *p* < 0.05, ** *p* < 0.005, and *** *p* < 0.0005; ns, not significant.

A decline in NAD^+^ levels can occur due to a defect in the enzymes responsible for synthesizing NAD^+^ or an increase in the enzymes that consume NAD^+^ (29). We assessed the mRNA and protein levels of both NAD^+^-synthesizing enzymes and NAD^+^-consuming enzymes (**Figure 2**). Since most cells generate NAD^+^ via the salvage pathways (30–32), we assessed the mRNA levels of several genes involved in the Preiss-Handler and salvage pathways. qPCR analysis did not show any genes with decreased expression within the intestinal epithelium of DSS mice as compared to control mice, although several genes showed significantly increased expression (**Figure 2A**). We further assessed the protein levels of several key enzymes involved in NAD^+^ synthesis from all three pathways and found no significant decrease in their levels in DSS tissue (**Figures 2B, C**). We next assessed the gene expression of NAD^+^-consuming enzymes, which included ADP-ribose polymerases (e.g., poly(ADP-ribose) polymerase-1 (*Parp1*) and *Parp2*), NAD^+^-dependent protein deacetylases (e.g., sirtuins), and the ecto-enzyme *Cd38* (Cluster of Differentiation 38). We found a significant increase in the levels of *Cd38*, *Sirt4*, *Sirt5*, and *Sirt6*, as well as a 2-fold increase in the expression of *Parp1* (**Figure 2D**). However, there was no significant difference in the protein levels of PARP1 in DSS intestinal tissue as compared to control intestine (**Figures 2E, F**). Since the *Parp1* gene is constitutively expressed, PARP1 activation cannot be deduced solely via protein expression, but instead by an increase in its enzymatic end-product, poly(ADP) ribose (pADPr) polymers (33). Therefore, we assessed the levels of pADPr-modified proteins by western blot and found a significant increase in the intestinal tissue from mice subjected to DSS as compared to the intestinal tissue from control mice (**Figures 2G, H**). These data demonstrate that the decrease in NAD^+^ levels within the intestinal epithelium during inflammation is a result of increased activity of NAD^+^-consuming proteins, including PARP1.

### NR supplementation during DSS colitis restores PGC1α levels and mitochondrial function

**Figure 3:**
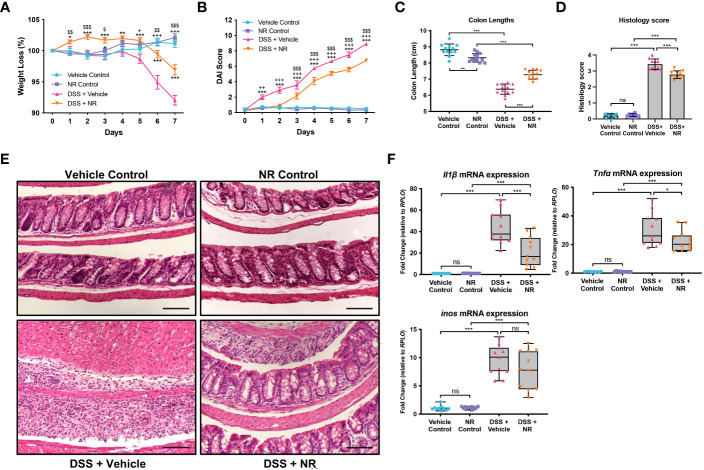
NAD^+^ supplementation improved disease in mice subjected to DSS. Acute colitis was induced by giving mice 2% DSS in their water for 7 days. Mice were treated with nicotinamide riboside (NR; 500 mg/kg/day) or vehicle (sterilized water; same mg/kg volume as for NR) once a day during DSS exposure. **(A)** The percent weight loss of each mouse was calculated and averaged as a group. **(B)** Disease activity index (DAI) scores were calculated for each mouse daily and averaged as a group. For A and B, the data are shown as the mean ± SEM; n=12/group. Asterisks (*) indicate a significance difference between DSS+Vehicle vs. DSS+NR groups via a two-way ANOVA. Plus signs (+) indicate a significant difference between Vehicle Control vs. DSS+Vehicle groups via a two-way ANOVA. Dollar signs ($) indicate a significance difference between NR Control vs. DSS+NR groups via a two-way ANOVA. **(C)** Colon lengths were measured (n=12/group). **(D, E)** Colonic sections were stained via H&E and scored in a blinded manner by a pathologist (n=8 for Vehicle Control and NR Control groups; n=10 for DSS+Vehicle and DSS+NR groups); scale bar = 100 μM. **(F)** The mRNA levels of proinflammatory cytokines were assessed via qPCR (n=10 for Vehicle Control and NR Control groups; n=11 for DSS+Vehicle and DSS+NR groups). Unless indicated otherwise, the data are shown as the mean ± SD. The data are representative of at least two independent experiments. Significance differences between groups [as determined by a one-way ANOVA **(C, D, F)** or two-way ANOVA **(A, B)**] are indicated on the graphs. * *p* < 0.05, ** *p* < 0.005, and *** *p* < 0.0005; ns, not significant.

Our data indicate that decreased NAD^+^ levels are responsible for decreased SIRT1 and PGC1α levels and increased mitochondrial dysfunction during DSS colitis. As such, we hypothesized that treating mice with an NAD^+^ precursor may restore both PGC1α levels and activity and mitochondrial function. Thus, we simultaneously subjected mice to DSS for 7 days and treated them either with the NAD^+^ precursor, NR, or the vehicle (water) via oral gavage. We found that mice subjected to DSS and treated with NR lost significantly less weight and had significantly lower DAIs as compared to mice subjected to DSS and treated with water (NR vehicle) (**Figures 3A, B**). Additionally, the colon lengths of DSS+NR mice (7.267 cm ± 0.3172 cm) were significantly longer as compared to DSS+Vehicle mice (6.475 cm ± 0.2864 cm), indicating lower levels of colonic inflammation (**Figure 3C**). Furthermore, histology demonstrated dramatically worse inflammatory changes and destruction of the intestinal architecture in DSS+Vehicle mice as compared to DSS+NR mice (histology scores 3.430 ± 0.3302 *vs*. 2.770 ± 0.2306, respectively; *p* < 0.0005) (**Figures 3D, E**). We also evaluated the mRNA levels of proinflammatory cytokines within the intestinal epithelium and found increased expression of *Nos2*, *Il1b*, and *Tnf* in both DSS treatment groups as compared to their respective controls, but decreased expression of *Il1* and *Tnf* in the DSS+NR group as compared to the DSS+Vehicle group, indicating that NR treatment did alleviate inflammation during experimental colitis (**Figure 3F**). Strikingly, in a therapeutic setting, NR supplementation also effectively lessened DSS-induced weight loss, DAI scores, and colonic shortening when given during recovery from DSS (**Figures S1E-G**).

**Figure 4:**
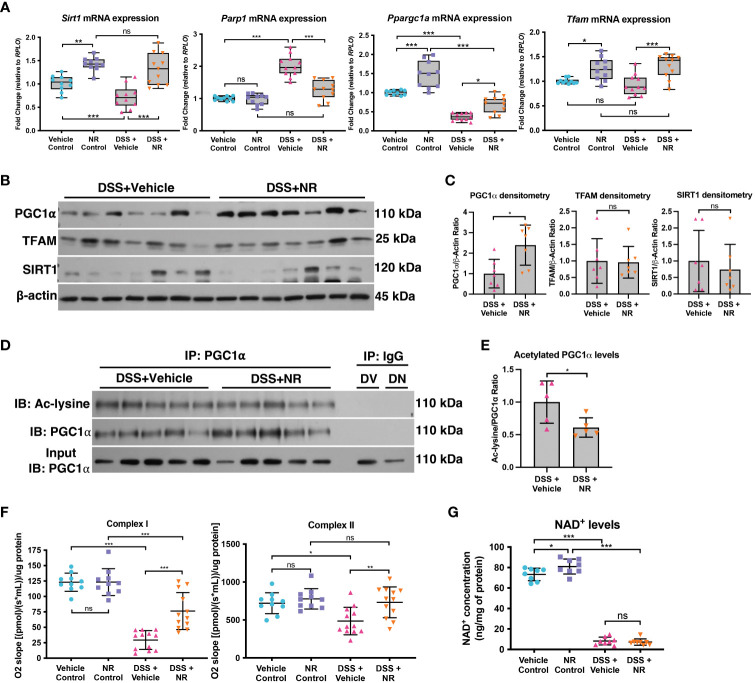
NAD^+^ supplementation preserved PGC1α activity and mitochondrial function in mice subjected to DSS. Mice subjected to DSS were treated with nicotinamide riboside (NR; 500 mg/kg/day) or vehicle (sterilized water; same mg/kg volume as for NR) once a day during DSS exposure. **(A)** The mRNA levels of genes were assessed via qPCR (n=10 for Vehicle Control and NR Control; n=11 for DSS+Vehicle and DSS+NR). **(B, C)** Protein levels of PGC1α, TFAM, and SIRT1 in the intestinal epithelium of DSS+Vehicle vs. DSS+NR mice (n=7/group). **(D, E)** Levels of acetylated PGC1α were assessed via immunoprecipitation (IP) and immunoblot (IB); DV, DSS+Vehicle; DN, DSS+NR. **(F)** Complex I and II activity within the colonic mucosa from control (n=10/group) vs. experimental mice (n=12/group). **(G)** NAD^+^ levels were evaluated using a NAD^+^/NADH Quantification Kit. Unless indicated otherwise, the data are shown as the mean ± SD. The data are representative of at least two independent experiments. Significance differences between groups [as determined by a one-way ANOVA **(A, F, G)** or unpaired two-tailed *t* test **(C, E)**] are indicated on the graphs. * *p* < 0.05, ** *p* < 0.005, and *** *p* < 0.0005; ns, not significant.

In order to assess if NR treatment restores the SIRT1-PGC1α axis during experimental colitis, we assessed the expression levels of SIRT1, PGC1α, and TFAM in DSS+NR and DSS+Vehicle mice. We found that the mRNA levels of *Sirt1*, *Ppargc1a*, and *Tfam* were not only increased in control mice treated with NR as compared to vehicle control mice but were also increased in DSS+NR mice as compared to DSS+Vehicle mice (**Figure 4A**). The protein levels of PGC1α were increased within the intestinal epithelium of DSS+NR mice, while the protein levels of TFAM and SIRT1 remained unchanged (**Figures 4B, C**). Furthermore, the levels of acetylated (inactive) PGC were decreased in DSS+NR mice as compared to DSS+Vehicle mice (**Figures 4D, E**). We and others have shown evidence of decreased activity of the electron transport chain during DSS colitis (5, 34, 35). To assess the activity of the electron transport chain in our model, we performed high-resolution respirometry using an Oroboros Oxygraph-2K on the intestinal epithelium from control mice and mice undergoing DSS with or without NR treatment. We found that the activity of Complex I and II were significantly decreased in DSS+Vehicle mice as compared to Vehicle Control mice (**Figure 4F**). Moreover, we also observed increased Complex I and II activity in DSS+NR mice as compared to DSS+Vehicle mice, indicating that NR treatment restored the NAD^+^ levels enough to increase mitochondrial function (**Figure 4F**). However, while NR treatment allowed for increases in mitochondrial function and PGC1α levels, it did not result in a significant or prolonged increase of cellular NAD^+^ levels within the intestinal epithelium of DSS mice (**Figure 4G**), which may be due to the short treatment period and rapid turnover of NAD^+^ during ongoing inflammation.

Since bacteria are known to metabolize NAD^+^ and have been shown to enhance the metabolism of oral NR supplementation in mice (36), we assessed the fecal microbiome via 16S rRNA sequencing. We observed differences in the alpha and beta diversity between the control and DSS groups, but we found no differences in the microbiome of DSS+NR and DSS+Vehicle groups, which indicated that NR supplementation did not alter the microbiome (**Figures S1A, B**). Furthermore, although there was a significant decrease in the *Parp1* mRNA levels in DSS+NR mice as compared to DSS+Vehicle mice (**Figure 4A**), there was no difference in the levels of pADPr-modified proteins in DSS+Vehicle and DSS+NR mice (**Figures S1C, D**), suggesting that NR supplementation did not reduce the levels of NAD^+^-consuming enzymes, such as PARP1, but instead transiently increased the NAD^+^ levels within the intestinal epithelium to allow for the activation of PGC1α. Our results demonstrate that NR supplementation during DSS colitis mitigated disease pathogenesis by increasing PGC1α levels and mitochondrial function.

### NR treatment does not ameliorate experimental colitis in *Pgc1α^ΔIEC^* mice

**Figure 5:**
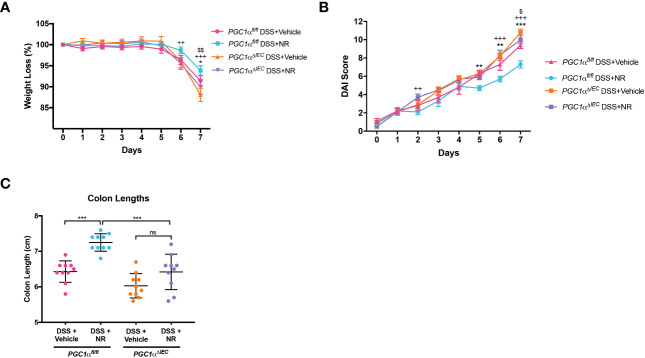
*Villin-cre;Pgc1α^fl/fl^* mice (*Pgc1α^ΔIEC^*) mice do not benefit from NR treatment during DSS colitis. Acute colitis was induced by giving mice 2% DSS in their water for 7 days. Mice were treated with nicotinamide riboside (NR; 500 mg/kg/day) or vehicle (sterilized water; same mg/kg volume as for NR) once a day during DSS exposure. **(A)** The percent weight loss of each mouse was calculated and averaged as a group (n=10/group). **(B)** Disease activity index (DAI) scores were calculated for each mouse daily and averaged as a group (n=10/group). For A and B, the data are shown as the mean ± SEM. Asterisks (*) indicate a significance difference between *Pgc1α^fl/fl^* DSS+Vehicle vs. *Pgc1α^fl/fl^* DSS+NR via a two-way ANOVA. Plus signs (+) indicate a significance difference between *Pgc1α^fl/fl^* DSS+NR vs. *Pgc1α^ΔIEC^* DSS+NR via a two-way ANOVA. Dollar signs ($) indicate a significance difference between *Pgc1α^fl/fl^* DSS+Vehicle vs. *Pgc1α^ΔIEC^* DSS+Vehicle. **(C)** Colon lengths were measured (n=10/group; mean ± SD). The data are representative of at least two independent experiments. Significance differences between groups [as determined by a one-way ANOVA **(C)** or two-way ANOVA **(A, B)**] are indicated on the graphs. * *p* < 0.05, ** *p* < 0.005, and *** *p* < 0.0005; ns, not significant.

We have previously shown a decrease in PGC1α expression and activity levels, as well as mitochondrial function, within the intestinal epithelium of mice undergoing experimental colitis (5). Moreover, we have also demonstrated that *Pgc1α^ΔIEC^* mice develop worse colitis when subjected to DSS as compared to *Pgc1α^fl/fl^ mice* (5). Here, we show that NR treatment during experimental colitis not only restores PGC1α levels (**Figures 4A-C**), but also Complex I and II activity (**Figure 4F**). To determine whether PGC1α is central to the disease improvements seen with NR supplementation during DSS colitis, we subjected *Pgc1α^ΔIEC^* mice (mice lacking PGC1α within the intestinal epithelium) and their WT littermates (*Pgc1α^fl/fl^*) to DSS and treated them with either NR or vehicle via oral gavage. We found that *Pgc1α^ΔIEC^* mice subjected to DSS+Vehicle had significantly more weight loss, higher DAI scores, and shorter colons at Day 7 as compared to *Pgc1α^fl/fl^* mice subjected to DSS+Vehicle (**Figures 5A-C**). Interestingly, however, the percent weight loss, DAI scores, and colon lengths of *Pgc1α^ΔIEC^* mice subjected to DSS+NR did not differ from *Pgc1α^ΔIEC^* mice subjected to DSS+Vehicle, whereas *Pgc1α^fl/fl^* mice subjected to DSS+NR lost significantly less weight, had longer colons, and exhibited significantly lower DAI scores at Day 7 as compared to *Pgc1α^fl/fl^* mice subjected to DSS+Vehicle (**Figures 5A-C**). These results suggest that the effects of NR require an intact PGC1α axis.

### The SIRT1-PGC1α axis is disrupted in infectious colitis

The *C. rodentium* model of infectious colitis has been utilized to assess mechanisms underlying the pathogenesis of IBD, as the mucosal immune response and pathology resulting from *C. rodentium* infection are similar to both human UC (37–39) and other models of experimental colitis (40). Moreover, the *C. rodentium* infectious colitis model has previously been shown to result in mitochondrial dysfunction and metabolic reprogramming (41–45). Thus, to validate our findings from the DSS colitis model and gain further insight into the importance of the SIRT1-PGC1α axis in colonic inflammation, we employed the *C. rodentium*-induced murine model of infectious colitis. We established the infectious colitis model by orally infecting mice with 1×10^9^ colony-forming units (CFUs) of *C. rodentium* (**Figure S2A**). We found that mice infected with *C. rodentium* lost significantly more weight (**Figure S2B**), had higher DAI scores (**Figure S2C**), and shorter colons (**Figure S2D**) as compared to Sham-infected mice, indicating that the infectious colitis model was successfully established. Additionally, the fecal stool burden of *C. rodentium* progressively increased over the duration of the model, peaking at Day 8 (**Figure S2E**). Histology demonstrated dramatically worse colitis in *Citrobacter*-infected mice, as evidenced by colonic hyperplasia and lamina propria inflammation (**Figures S2F, G**). These results show that we successfully established the *C. rodentium* model of infectious colitis.

**Figure 6:**
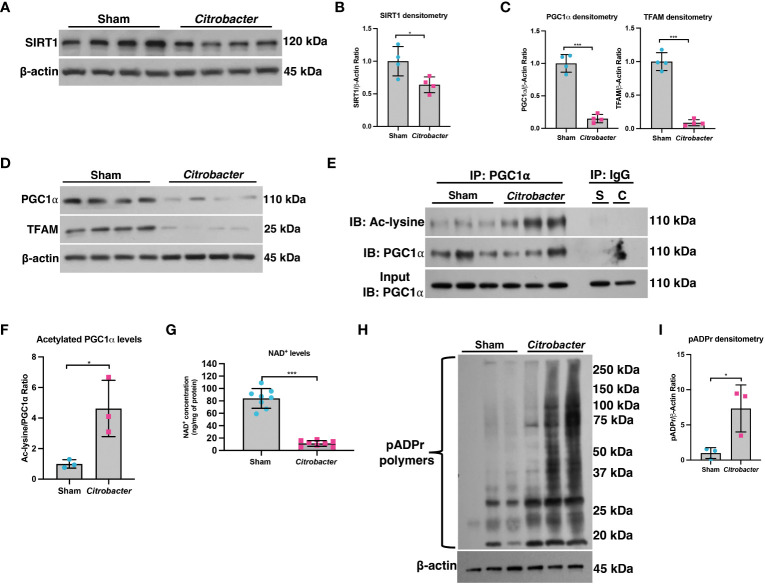
The SIRT1-PGC1α axis is disrupted during infectious colitis. Infectious colitis was induced by infecting mice with 1×10^9^ colony-forming units (CFUs) of *Citrobacter rodentium.* **(A, B)** The protein levels of SIRT1 and **(C, D)** PGC1α and TFAM in the intestinal epithelium of Sham- and *Citrobacter*-infected mice (n=4/group). **(E, F)** Levels of acetylated PGC1α were assessed via immunoprecipitation (IP) and immunoblot (IB); S, Sham-infected; C, *Citrobacter*-infected. **(G)** NAD^+^ levels were evaluated using a NAD^+^/NADH Quantification Kit. **(H, I)** Western blot analysis of poly(ADP)-ribose (pADPr)-modified proteins within the intestinal epithelium of mice subjected to DSS as compared to control mice (n=3/group). Unless indicated otherwise, the data are shown as the mean ± SD. The data are representative of at least two independent experiments. Significance differences between groups (as determined by a two-tailed *t* test) are indicated on the graphs. * *p* < 0.05 and *** *p* < 0.0005; ns, not significant.

We then investigated the importance of the PGC1α axis during infectious colitis. We found a significant decrease in the SIRT1 protein levels in *Citrobacter*-infected mice as compared to Sham-infected mice (**Figures 6A, B**). We further found a significant reduction in the protein levels of PGC1α and TFAM in *Citrobacter*-infected mice (**Figures 6C, D**), as well as an increase in the levels of acetylated PGC1α (**Figures 6E, F**). We then assessed the levels of NAD^+^ within the intestinal epithelium and found a dramatic reduction in the levels of NAD^+^ within the intestinal epithelium of *Citrobacter*-infected mice as compared to Sham-infected mice (**Figure 6G**). Thus, *C. rodentium* infection also resulted in reduced NAD^+^ levels within the intestinal epithelium, which would account for the decreased expression of SIRT1 and increased pool of acetylated PGC1α. Finally, we evaluated the activity of PARP1 and found a substantial increase in the levels of pADPr-modified proteins in *Citrobacter*-infected mice by western blot (**Figures 6H, I**), indicating that infection with *C. rodentium* induces PARP1 activity.

### NR supplementation restores PGC1α levels and mitochondrial function during infectious colitis

**Figure 7:**
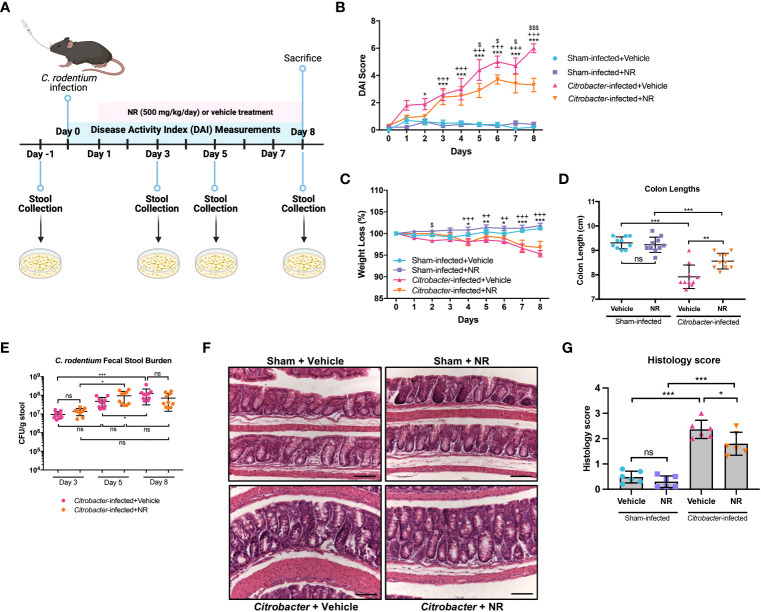
NAD^+^ supplementation improved disease in *Citrobacter*-infected mice. Mice were treated with nicotinamide riboside (NR; 500 mg/kg/day) or vehicle (sterilized water; same mg/kg volume as for NR) once a day starting one day post-infection. **(A)** A schematic outlining the infectious colitis model used in this study. Image was created with BioRender.com. **(B)** Disease activity index (DAI) scores were calculated for each mouse daily and averaged as a group. **(C)** The percent weight loss of each mouse was calculated and averaged as a group. For B and C, the data are shown as the mean ± SEM; n=10/group. Asterisks (*) indicate a significance difference between Sham+Vehicle vs. *Citrobacter*+Vehicle groups via a two-way ANOVA. Plus signs (+) indicate a significant difference between Sham+NR vs. *Citrobacter*+NR groups via a two-way ANOVA. Dollar signs ($) indicate a significance difference between *Citrobacter+*Vehicle vs. *Citrobacter*+NR groups via a two-way ANOVA. **(D)** Colon lengths were measured (n=10/group). **(E)** The fecal stool burden (CFU/g of stool) of *C rodentium* was determined by plating (n=10/group). **(F, G)** Colonic sections were stained via H&E and scored in a blinded manner by a pathologist (n=6/group); scale bar = 100 μM. Unless indicated otherwise, the data are shown as the mean ± SD. The data are representative of at least two independent experiments. Significance differences between groups [as determined by a one-way ANOVA **(D, G)** or two-way ANOVA **(B, C, E)**] are indicated on the graphs. * *p* < 0.05, ** *p* < 0.005, and *** *p* < 0.0005; ns, not significant.

We next treated mice with NR (**Figure 7A**) to determine if it could restore PGC1α levels and mitochondrial health. We found that *Citrobacter*+NR mice had significantly lower DAIs (**Figure 7B**) as compared to *Citrobacter*+Vehicle mice. Also, mice infected with *C. rodentium* and treated with NR trended towards less weight loss (**Figure 7C**) and had significantly longer colons (**Figure 7D**) as compared to mice infected with *C. rodentium* and treated with Vehicle, indicating lower levels of colonic inflammation. Additionally, while the fecal stool burden of *Citrobacter*+Vehicle mice progressively increased over the duration of the model and peaked at Day 8 post-infection (average 1.24 × 10^8^ CFU/g stool), similar to our prior data (**Figure S2E**), the fecal stool burden of *Citrobacter*+NR mice increased and peaked at Day 5 post-infection (average 9.43 × 10^7^ CFU/g stool) and exhibited a slightly lower, but not statistically significant average CFU count on Day 8 post-infection (7.02 × 10^7^ CFU/g stool) as compared to *Citrobacter*+Vehicle mice (**Figure 7E**). Furthermore, *Citrobacter*+NR mice had milder hyperplasia and lower levels of immune cell infiltration into the lamina propria compared to *Citrobacter*+Vehicle mice as evidenced by H&E staining (histology score 1.800 ± 0.4561 *vs*. 2.367 ± 0.3615, respectively; *p* < 0.05) (**Figures 7F, G**). Taken together, mice infected with *C. rodentium* and treated with NR demonstrated improved disease activity and colonic inflammation as compared to mice infected with *C. rodentium* and treated with the vehicle.

**Figure 8:**
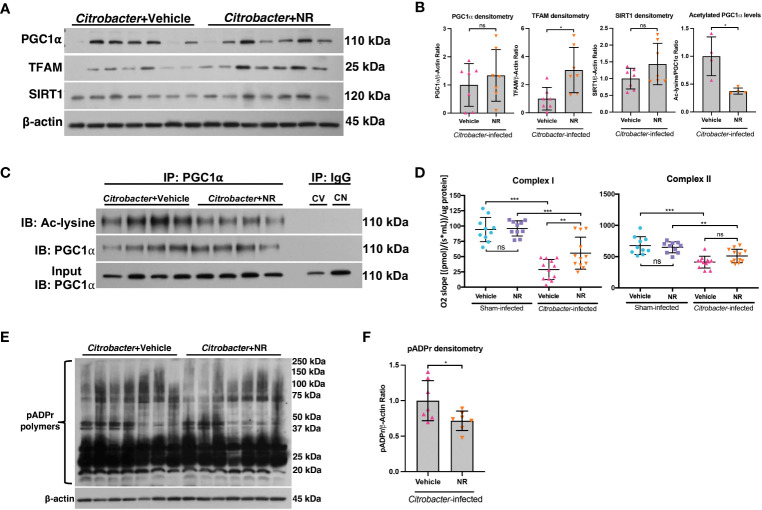
NAD^+^ repletion improved disease in a model of infectious colitis. Infectious colitis was induced by infecting mice with 1×10^9^ colony-forming units (CFUs) of *Citrobacter rodentium.* Mice were treated with nicotinamide riboside (NR; 500 mg/kg/day) or vehicle (sterilized water; same mg/kg volume as for NR) once a day starting one day post-infection. **(A, B)** The protein levels of PGC1α, TFAM, and SIRT1 in the intestinal epithelium of *Citrobacter*-infected mice treated with Vehicle or NR (n=7/group). **(B, C)** Levels of acetylated PGC1α were assessed via immunoprecipitation (IP) and immunoblot (IB); CV, *Citrobacter*+Vehicle; CN, *Citrobacter*+NR. **(D)** Complex I and II activity within the colonic mucosa from control vs. experimental mice (n=10/group for Sham+Vehicle and Sham+NR; n=12/group for *Citrobacter*+Vehicle and *Citrobacter*+NR). **(E, F)** Western blot analysis of poly(ADP)-ribose (pADPr)-modified proteins within the intestinal epithelium of *Citrobacter*+Vehicle mice as compared to *Citrobacter*+NR mice (n=7/group). Unless indicated otherwise, the data are shown as the mean ± SD. The data are representative of at least two independent experiments. Significance differences between groups [as determined by an unpaired two-tailed *t* test **(B, F)** or one-way ANOVA **(D)**] are indicated on the graphs. * *p* < 0.05, ** *p* < 0.005, and *** *p* < 0.0005; ns, not significant.

We demonstrated that NR treatment restored the SIRT1-PGC1α axis during DSS colitis and resulted in increased mitochondrial complex activity (**Figure 4**). To determine if NR treatment had similar effects on the SIRT1-PGC1α axis during infectious colitis, we assessed the SIRT1-PGC1α axis in mice infected with *C. rodentium* and treated with NR or Vehicle. We found increased protein levels of TFAM within the intestinal epithelium of *Citrobacter*+NR mice as compared to *Citrobacter*+Vehicle mice, but unchanged SIRT1 and PGC1α protein levels (**Figures 8A, B**). Furthermore, the levels of acetylated (inactive) PGC were decreased in *Citrobacter*+NR mice as compared to *Citrobacter*+Vehicle mice (**Figures 8B, C**). We also observed a significant increase in Complex I activity in *Citrobacter*+NR mice as compared to *Citrobacter*+Vehicle mice (**Figure 8D**). Interestingly, we also found a decrease in the levels of pADPr-modified proteins in *Citrobacter*-infected mice treated with NR as compared to *Citrobacter*-infected mice treated with vehicle (**Figure 8E, F**). In summary, our data demonstrate that NR treatment in mice with infectious colitis restores mitochondrial function, and surprisingly, reduces the activity of PARP1.

### The SIRT1-PGC1α axis is disrupted in human UC

**Figure 9:**
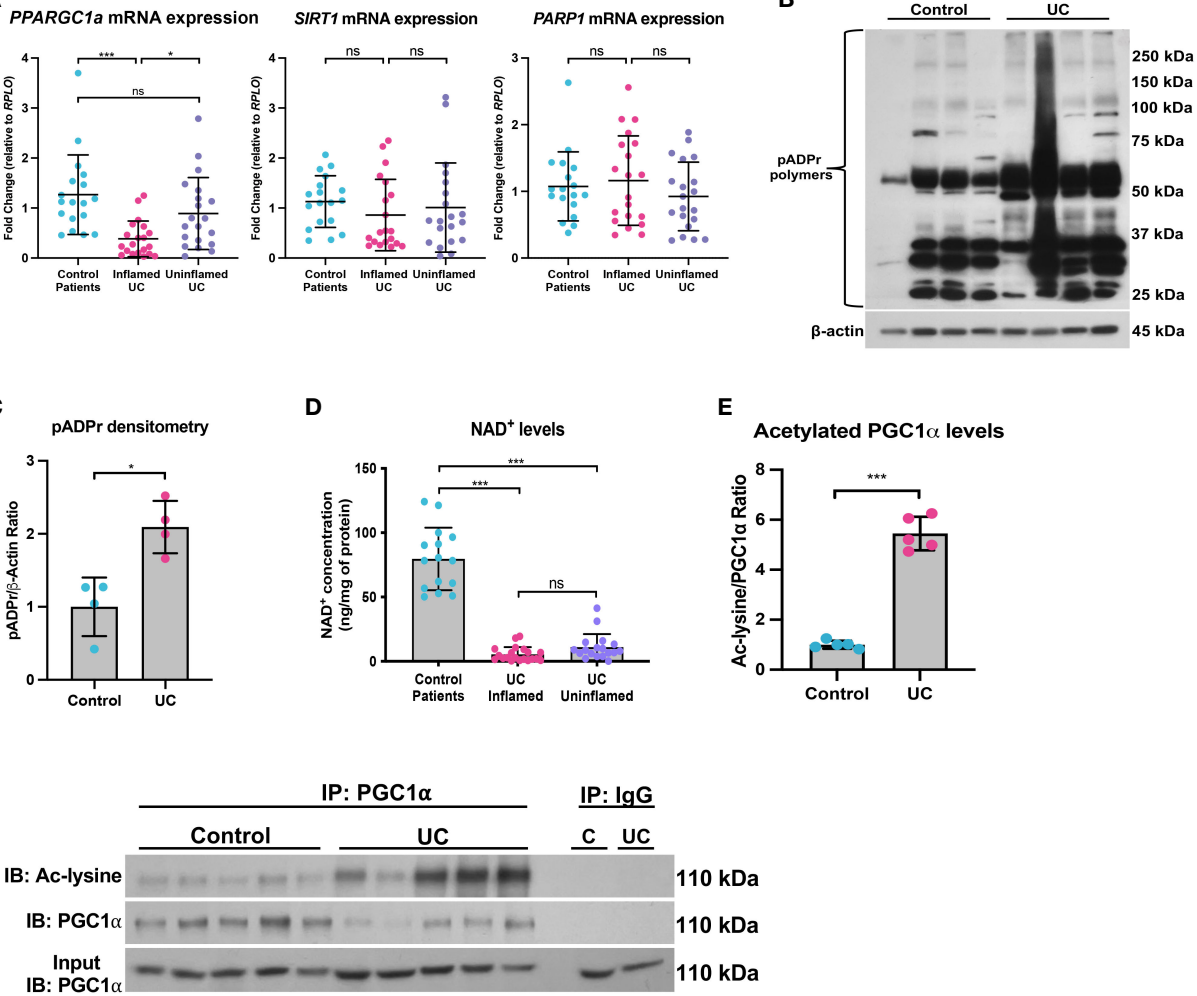
The SIRT1-PGC1α axis is disrupted during human colitis. Human intestinal tissue was collected from healthy control pediatric patients or pediatric patients with ulcerative colitis (UC). **(A)** The mRNA levels of genes were assessed via qPCR (n=18 for Control Patients; n=20 for Inflamed UC and Uninflamed UC). **(B, C)** Western blot analysis of poly(ADP)-ribose (pADPr)-modified proteins within the intestinal epithelium of Healthy Controls vs. UC patients (n=4/group). **(D)** NAD^+^ levels were evaluated using a NAD^+^/NADH Quantification Kit. **(E, F)** Levels of acetylated PGC1α were assessed via immunoprecipitation (IP) and immunoblot (IB); C, Control; UC, ulcerative colitis. Unless indicated otherwise, the data are shown as the mean ± SD. The data are representative of at least two independent experiments. Significance differences between groups [as determined by an unpaired two-tailed *t* test **(C, E)** or one-way ANOVA **(A, D)**] are indicated on the graphs. * *p* < 0.05 and *** *p* < 0.0005; ns, not significant.

We and others have previously shown that the mRNA and protein levels of PGC1α are downregulated in the intestine of adult and pediatric IBD patients (5, 11). Here, we demonstrate that the mRNA levels of *PPARGC1a* are downregulated in inflamed mucosal biopsy samples from pediatric patients with UC as compared to control mucosal biopsies and uninflamed mucosal biopsies from pediatric UC patients (**Figure 9A**). We also found that the while mRNA expression of *SIRT1* trended down in inflamed mucosal biopsy samples, there was no significant difference in *PARP1* expression (**Figure 9A**). However, since, the *PARP1* gene is constitutively expressed, we further assessed the activity of PARP1 in UC patients as compared to healthy controls and found a significant increase in the levels of pADPr-modified proteins within the intestinal epithelium of UC patients (**Figures 9B, C**), implying that PARP1 is activated during UC.

Since NAD^+^ is intimately connected to a functional SIRT1-PGC1α axis, we then assessed the NAD^+^ levels in mucosal biopsy samples from control and UC patients. We found a significant decrease in the NAD^+^ levels in inflamed and uninflamed mucosal biopsy samples from pediatric patients with UC as compared to control mucosal biopsies (**Figure 9D**), which suggests that NAD^+^ depletion may occur in the colons of UC patients well before the manifestation of inflammation, or that NAD^+^ metabolism may remain dysregulated within the intestinal epithelium after inflammation has subsided. We also show an increase in the levels of acetylated (inactive) PGC1α within the intestinal epithelium of patients with UC as compared to control patients (**Figure 9E, F**), indicating that not only are the levels of PGC1α decreased, but the activity of the remaining protein is also decreased. Taken together, our data demonstrate that the NAD^+^ depletion within the intestinal epithelium may be the underlying cause of decreased PGC1α expression and mitochondrial function during UC.

## Discussion

**Figure 10:**
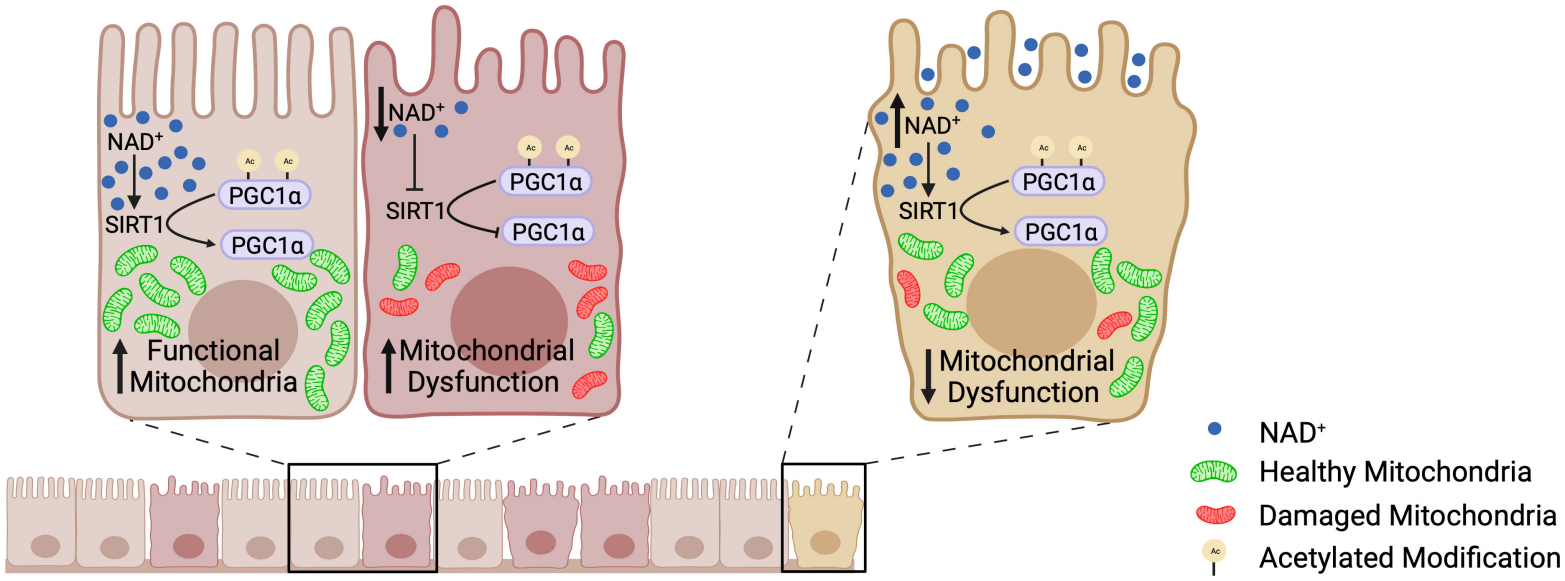
The SIRT1-PGC1α axis during intestinal inflammation. At homeostasis, NAD^+^ levels are adequate such that SIRT1 is able to deacetylate and activate PGC1α, resulting in mitochondrial biogenesis and normal mitochondrial function. However, during intestinal inflammation (e.g., experimental colitis or ulcerative colitis), NAD^+^ levels are depleted within the intestinal epithelium by the activation of PARP1, which renders SIRT1 unable to activate PGC1α. This results in decreased mitochondrial biogenesis and subsequent mitochondrial dysfunction. Mitochondrial dysfunction within the intestinal epithelial cells has detrimental effects on the barrier integrity of the intestinal epithelium. Our preclinical studies have shown that oral supplementation with the NAD^+^ precursor, nicotinamide riboside, increases the activation of PGC1α and overall mitochondrial health and function. Thus, future therapeutic approaches targeting the activity of PGC1α, and in turn mitochondrial health, may complement the treatment for IBD and improve outcomes in patients.

Until recently, little was known about mitochondrial function within the intestinal epithelium or how perturbations in mitochondrial function may contribute to IBD. Our laboratory was the first to define a role for decreased PGC1α expression and activity in the progression of inflammation during IBD (5). Colonic mitochondriopathy was later observed in patients suffering from pediatric-onset UC, with PGC1α downregulation as a signature feature of treatment-naïve UC, correlating with disease severity (11). However, the process driving downregulation of PGC1α expression and activity during IBD remains poorly outlined. Herein, we utilized a combination of murine models of colitis, human UC biopsy and surgical tissue, and high-resolution respirometry to delineate the mechanism underlying the increased levels of acetylated (inactive) PGC1α within the intestinal epithelium during colonic inflammation. We found that NAD^+^ depletion within the intestinal epithelium during inflammation decreases the expression and activity of SIRT1, resulting in the inactivation of PGC1α via increased acetylation and a subsequent decrease in mitochondrial function (**Figure 10**).

PGC1α is known to be deacetylated and activated by the NAD^+^-dependent deacetylase, SIRT1 (12, 13). Similar to human UC, we found that both the mRNA and protein levels of SIRT1 were significantly decreased within the intestinal epithelium of mice undergoing experimental colitis as compared to control mice (22, 23). SIRT1 is a redox-sensitive protein and is susceptible to reversible and irreversible modifications evoked by ROS and RNS, influencing both the expression and activity of SIRT1 (46–48). Although oxidative stress is considered to be a critical factor in the pathogenesis of experimental colitis in mice (5, 49–51), we showed no difference in the activity of SIRT1 from control and DSS-subjected mice when immunoprecipitated from colonic mucosal scrapings and provided with ample NAD^+^. This suggests that SIRT1 did not undergo irreversible modifications that could have decreased its expression and activity *in vivo*. SIRT1 activity is also known to be intimately regulated by cellular NAD^+^, and several studies have reported fluctuations in SIRT1 expression that correlate with NAD^+^ levels (26–28). Here, we found significantly reduced levels of NAD^+^ within the colonic scrapings from DSS mice as compared to control mice. These results suggest that decreased expression of SIRT1 and PGC1α stem, at least in part, from a pathologic depletion of NAD^+^ levels within the intestinal epithelium during colitis.

Homeostasis of NAD^+^ levels depends on a balance between NAD^+^ biosynthesis and NAD^+^ consumption (29). We assessed the expression of several genes and proteins involved in NAD^+^ synthesis. While none of the tested NAD^+^ genes or proteins were significantly downregulated during experimental colitis, there was a significant increase in NAMPT, the rate-limiting enzyme in the NAD^+^ salvage pathway (52). This is consistent with previous studies, which have reported significant increases in NAMPT during both experimental colitis and human IBD. Additionally, Garner et al. reported that that inhibition of NAMPT via the small-molecule inhibitor FK866 effectively reduces NAD^+^ levels within the intestinal epithelium and ameliorates DSS-induced colitis by decreasing the expression of mucosal NAD^+^-consuming enzymes, including PARP1 and SIRT6, and reducing the mucosal immune cell infiltration and release of proinflammatory cytokines and chemokines (53). However, while Garner et al. concluded that NAD^+^ fuels intestinal inflammation (53), our results indicate that decreased mucosal NAD^+^ levels contribute to intestinal inflammation. These contradictory results may be due to the different models used or potential off-target effects of the chemical inhibitor FK866. For instance, FK866 not only affects the function of intracellular NAMPT, which is needed for NAD^+^ biosynthesis, but may also influence extracellular NAMPT (eNAMPT)—an enzymatically active extracellular form of NAMPT secreted by various cells. eNAMPT is considered to be a damage-associated molecular pattern and to possess cytokine-like effects on the immune system (54, 55). eNAMPT has been reported to be increased in IBD and to correlate with disease severity (56–61). Serum eNAMPT levels have been shown to be elevated in patients who fail to respond to anti-TNFα therapy (56). Further, neutralization of eNAMPT ameliorates experimental colitis (56). Interestingly, FK866 did not alter mucosal NAD^+^ or ATP levels at steady state or mucosal ATP levels during disease (53), suggesting that the intestinal epithelial cells produced energy via an NAMPT-independent pathway. Nonetheless, detailed effects of NAMPT inhibition or eNAMPT neutralization on the SIRT1-PGC1α axis and mitochondrial function during intestinal inflammation have not yet been reported.

Conversely, we provide evidence that NAD^+^ depletion within the intestinal epithelium during inflammation results from increased activity of NAD^+^-consuming enzymes. pADPr, cyclic ADPr, O-acyl-ADPr, and nicotinamide are degradation products resulting from the breakdown of NAD^+^ by NAD^+^-consuming enzymes (62). Nicotinamide and ADPr levels were previously shown to be increased in inflamed UC patients (63, 64). Here, we show an increase in pADPr-modified proteins—an indication of increased PARP activity—in mice undergoing experimental colitis. Among the 18 members of the PARP family, PARP1 is the most abundant isoform and responsible for over 90% of global pADPr synthesis following DNA damage (65–67). PARP1 is one of the few PARP family members capable of true ADPR polymerization, as the majority can only transfer a single ADPr (67). Upon activation by DNA damage, PARP1 covalently modifies itself and other nuclear proteins via poly(ADP-ribosyl)ation (PARylation) (67). PARP1 is heavily dependent on NAD^+^ for its activity and can deplete cellular energy stores under conditions of excessive activation (15, 66). Oxidative damage during the pathogenesis of IBD is known to promote structural alterations in DNA (49–51), and PARP1 activation has been shown to be increased within the intestinal mucosa of mice subjected to experimental colitis and in humans with UC (68–71). Interestingly, PARP1 activation is known to dysregulate SIRT1 activity via NAD^+^ depletion during skeletal muscle fatigue, aging, and cerebral ischemia, and deletion of PARP1 results in increased SIRT1 activity, higher mitochondrial content, and increased energy expenditure (72–76). Moreover, genetic deficiency or pharmacological inhibition of PARP1 reduces colitis in rodents (71, 76–79); however, no study has assessed the link between PARP1 and NAD^+^ levels during colitis. Our findings suggest that activation of PARP1 may be primarily responsible for the depletion in cellular NAD^+^ stores and eventual mitochondrial dysfunction that occurs within the intestinal epithelium during colitis.

Although we provide evidence of a PARP-related decline in NAD^+^ levels during colitis, we cannot rule out the role CD38 may also play in modulating NAD^+^ levels during intestinal inflammation. CD38, an NAD^+^ glycohydrolase, is predominately expressed on immune cells, including resident and infiltrating immune cells in the colonic mucosa of human and mice, as well as T lymphocytes within the lamina propria of the human intestine (80, 81). Upregulation of CD38 protein expression has been observed in the inflamed regions of the colonic mucosa from patients with UC (82–86). Moreover, CD38^-/-^ mice subjected to DSS colitis only developed mild colitis as compared to WT mice (83). Our results show an increase in *Cd38* gene expression in the mucosal scrapings of mice undergoing DSS-induced colitis. It is important to note that intestinal mucosal scrapings are an enriched sample of intestinal epithelial cells, and as such, may also contain other cell types, including immune cells. A previous study, however, demonstrated that mucosal scrapings are a valid method for obtaining a representative sample of intestinal epithelial cells (87), as the mucosal scrapings contained 94.2% ± 2.1% epithelial cells, 0.56% ± 0.1% CD11c^+^ cells, 1.21% ± 0.1% CD45R^+^ cells, and 2.48% ± 0.4% F4/80^+^ cells (84). Nonetheless, further studies are needed to confirm the protein expression and activity of CD38, as well as its underlying role specifically within the intestinal epithelium during inflammation.

Several studies have previously reported amelioration of inflammation in animal models by NAD^+^ repletion (88–92). As such, we treated mice undergoing experimental colitis (both DSS colitis and infectious colitis) with the NAD^+^ precursor, NR, and assessed the effects of NR treatment on the SIRT1-PGC1α axis. We found that NR treatment lessened disease severity in DSS models of colitis in both a preventative and therapeutic manner. NR treatment not only reduced DAI scores, but also increased the expression of PGC1α and TFAM, as well as the activity of Complex I and II in mice undergoing experimental colitis, indicating that NR treatment increased the NAD^+^ levels within the colonocytes. Interestingly, NR treatment did not reduce the activity of PARP1 in DSS colitis, as evidenced by similar levels of pADPr-modified proteins in the intestinal epithelium of DSS+Vehicle versus DSS+NR mice, but NR did unexpectedly reduce pADPr-modified protein levels in *Citrobacter*-infected mice. Moreover, we further confirmed that PGC1α is central to the disease improvements seen with NR supplementation during experimental colitis, as NR supplementation did not improve DAI scores in *PGC1α^ΔIEC^* mice subjected to DSS.

Although our results demonstrate positive effects of nicotinamide riboside (NR) supplementation on PGC1α and mitochondrial function during experimental colitis, it is perplexing that NR supplementation did not exhibit beneficial effects on the intestinal dysbiosis of mice undergoing experimental colitis. We surmise this may be because the length of NR supplementation used in our study, 7 days, was not long enough to induce changes at the microscopic level in the intestine. Several previously published studies, which report changes in the gut microbiota by utilizing NR to treat disease in various animal models, followed an experimental design with at least 4 weeks of NR treatment (36, 93–97). Studies that have demonstrated changes in the intestinal microbiota by treating various animal models with other NAD^+^ precursors (e.g., nicotinamide, nicotinamide mononucleotide, tryptophan) also followed a longer experimental model, with at least 2 weeks of treatment (98–105). Moreover, studies that have reported restoration of the gut microbiota via supplementation of NAD^+^ precursors in experimental colitis models also used a treatment period of at least 2 weeks (99, 100). Thus, the duration of NAD^+^ treatment during experimental colitis may need to be optimized to observe maximal therapeutic benefits, including reversing DSS-induced gut dysbiosis. We also found it interesting that, despite the restoration of the intestinal architecture, in addition to the observed increases in PGC1α expression and mitochondrial function within the intestinal epithelium of colitic mice after NR treatment, we did not see an increase in NAD^+^ levels within the intestinal epithelium. We speculated that the time between administering the last NR dose and measuring NAD^+^ levels (24 hours) might have been too long to capture the transient increase in NAD^+^ levels induced by NR. In fact, this is supported by previous studies, which showed that orally administered NR increases NAD^+^ levels in a biphasic manner (106, 107). NR is initially both absorbed directly by the small intestine and degraded to nicotinamide by the CD38 paralogue BST1 (bone marrow stromal cell antigen 1) in the small intestine. The colonic gut microbiota then metabolizes the NR-derived nicotinamide to nicotinic acid, which is then absorbed and used in the generation of NAD^+^ in at least the intestine and liver (106, 108). The early phase was reported to occur within one hour after NR administration and the late phase approximately three hours after NR administration, supporting our conclusion that measuring NAD^+^ levels within the intestinal epithelium at the conclusion of our experiment does not accurately reflect the increase in cellular NAD^+^ levels shortly after NR treatment (106, 108). It is also important to note that while DSS disrupted the intestinal microbiota, NR supplementation in control and DSS mice did not alter the composition or structure of the gut microbiota. Thus, while the current study demonstrates the feasibility of enhancing PGC1α expression and mitochondrial function in the intestinal epithelium by NR treatment, further studies are needed to delineate the effects of NR supplementation on NAD^+^ metabolism within the intestinal epithelium, as well as the lasting effects of NR supplementation on mitochondrial health and the intestinal microbiota during intestinal inflammation.

The introduction of biologic therapies, including anti-tumor necrosis factor (anti-TNF) inhibitors, has dramatically altered our approach to treating IBD in recent decades; however, up to 30% of patients do not respond to initial anti-TNF treatment (primary non-responder) and an additional 50% of responders will lose their response during treatment (secondary non-responders) (109–112). As such, alternatives are needed for those patients with refractory disease. In the current study, we show a significant depletion in intestinal epithelial NAD^+^ levels in mice undergoing experimental colitis and in pediatric patients with UC. We further demonstrate that NAD^+^ supplementation lessens disease severity in murine models of experimental colitis. Considering the significance that NAD^+^ potentially plays in the development and progression of IBD, NAD^+^ biosynthesis and metabolism are attractive therapeutic targets for the treatment of IBD. IBD is a multifactorial disease, and as such, we surmise that the optimal treatment will be one that targets multiple aspects of disease. While our results show that NAD^+^ precursor supplementation can lessen disease severity, we expect that the maximal benefits of NAD^+^ precursors against colitis would be exerted when combined with conventional pharmacologic therapy and biologic therapy currently used to treat disease. Further, we suspect that NAD^+^ precursor supplementation will work best with other NAD^+^-supporting therapeutics. Indeed, previous studies have shown that NAD^+^ supplementation combined with other nutraceutical interventions, such as coenzyme 10, resveratrol, ginsenoside, or pterostilbene, potentiate the effects of NAD^+^ supplementation alone (113, 114). These studies suggest that NAD^+^-nutraceutical combination therapy is beneficial, as nutraceutical interventions maximize the potential of NAD^+^ precursors, enhancing efficacy and exerting their own various health benefits.

It is important to note that NAD^+^ levels have also been shown to decline in the intestine with healthy aging (115–117). However, it is currently unknown if declining NAD^+^ levels within the intestine renders a person more susceptible to intestinal inflammation. Although often diagnosed in younger patients, up to 20% of IBD patients are over 65 years old (118). Research has showed that many aging-associated diseases can be slowed, or even possibly reversed, by restoring cellular NAD^+^ levels (119). Indeed, NAD^+^ supplementation has been shown to rejuvenate adult stem cells from aged mice *in vitro* (96), as well as improve the structure and function of the intestine in aging mice (117, 120). Although NAD^+^ supplementation has shown beneficial effects against murine colitis (present study (99, 100); and healthy intestinal aging (119), knowledge regarding the outcomes of administering NAD^+^ precursors for the prevention or treatment of IBD in the context of aging is limited (121). There are physiologic and phenotypic differences in geriatric-onset IBD, as compared to pediatric- and adult-onset IBD, that impact the therapeutic management of IBD in the elderly (122). Thus, further research is needed to reveal the primary drivers of NAD^+^ depletion throughout IBD development and progression in different patient age groups.

In conclusion, we demonstrate decreased NAD^+^ levels within the intestinal epithelium of mice undergoing experimental and humans with UC. Based on our results, we hypothesize that activation of the DNA repair enzyme, PARP1, within the intestinal epithelium depletes cellular NAD^+^ stores, which renders SIRT1 unable to activate PGC1α, consequently resulting in mitochondrial dysfunction. We further show that NAD^+^ repletion with oral NR supplementation decreases disease severity while increasing the expression of PGC1α and improving mitochondrial function. Importantly, the effects of NAD^+^ repletion required an intact PGC1α axis, underscoring the importance of PGC1α to the health and homeostasis of the intestinal epithelium. Our study suggests that restoring NAD^+^ levels specifically within intestinal epithelial cells is a promising strategy to improve mitochondrial and metabolic health. Further understanding of the drivers underlying NAD^+^ depletion within the intestinal epithelium may allow for the development of novel therapies to complement the treatment of IBD.

## Data availability statement

The 16S rRNA sequencing data presented in this study are deposited in the SRA repository (https://www.ncbi.nlm.nih.gov/sra), accession number PRJNA992773.

## Ethics statement

The studies involving humans were approved by University of Pittsburgh Institutional Review Board. The studies were conducted in accordance with the local legislation and institutional requirements. Written informed consent for participation in this study was provided by the participants’ legal guardians/next of kin. The animal study was approved by University of Pittsburgh IACUC. The study was conducted in accordance with the local legislation and institutional requirements. No potentially identifiable images or data are presented in this study.

## Author contributions

EN and KM conceived the project. EN designed the experiments and HM and KM contributed. EN, EC, HM, BG, DF, and MF acquired and interpreted data. EN and EC performed statistical analysis. EC acquired written informed consent from all patients and collected all human tissue specimens. BF, MR, and MM performed the 16S rRNA sequencing and analysis. QW performed the histological analysis. JP and CS provided technical and intellectual support. EN generated the figures and wrote the manuscript. EN, EC, HM, and KM critically revised the manuscript and provided important intellectual content. All authors reviewed and approved the final manuscript prior to submission. KM acquired funding for this project. All authors contributed to the article and approved the submitted version.
