# Supplementary material for: Epithelial NAD+ depletion drives mitochondrial dysfunction and contributes to intestinal inflammation
Source: Front Immunol. 2023 Sep 7;14:1231700. doi: 10.3389/fimmu.2023.1231700 (PMC10512956; doi:10.3389/fimmu.2023.1231700)
Supplement: Supplementary file 1 [file DataSheet_1.pdf]

Supplementary Material

Epithelial NAD^+ Depletion Drives Mitochondrial Dysfunction and Contributes to Intestinal Inflammation

Elizabeth A. Novak, Erin C. Crawford, Heather L. Mentrup, Brian D. Griffith, David M. Fletcher, Meredith R. Flanagan, Corinne Schneider, Brian Firek, Matthew B. Rogers, Michael J. Morowitz, Jon D. Piganelli, Qian Wang, Kevin P. Mollen[1*]

*** Correspondence:**
Kevin P. Mollen
Kevin.Mollen@chp.edu

Figure S1
Figure S2
Table S1

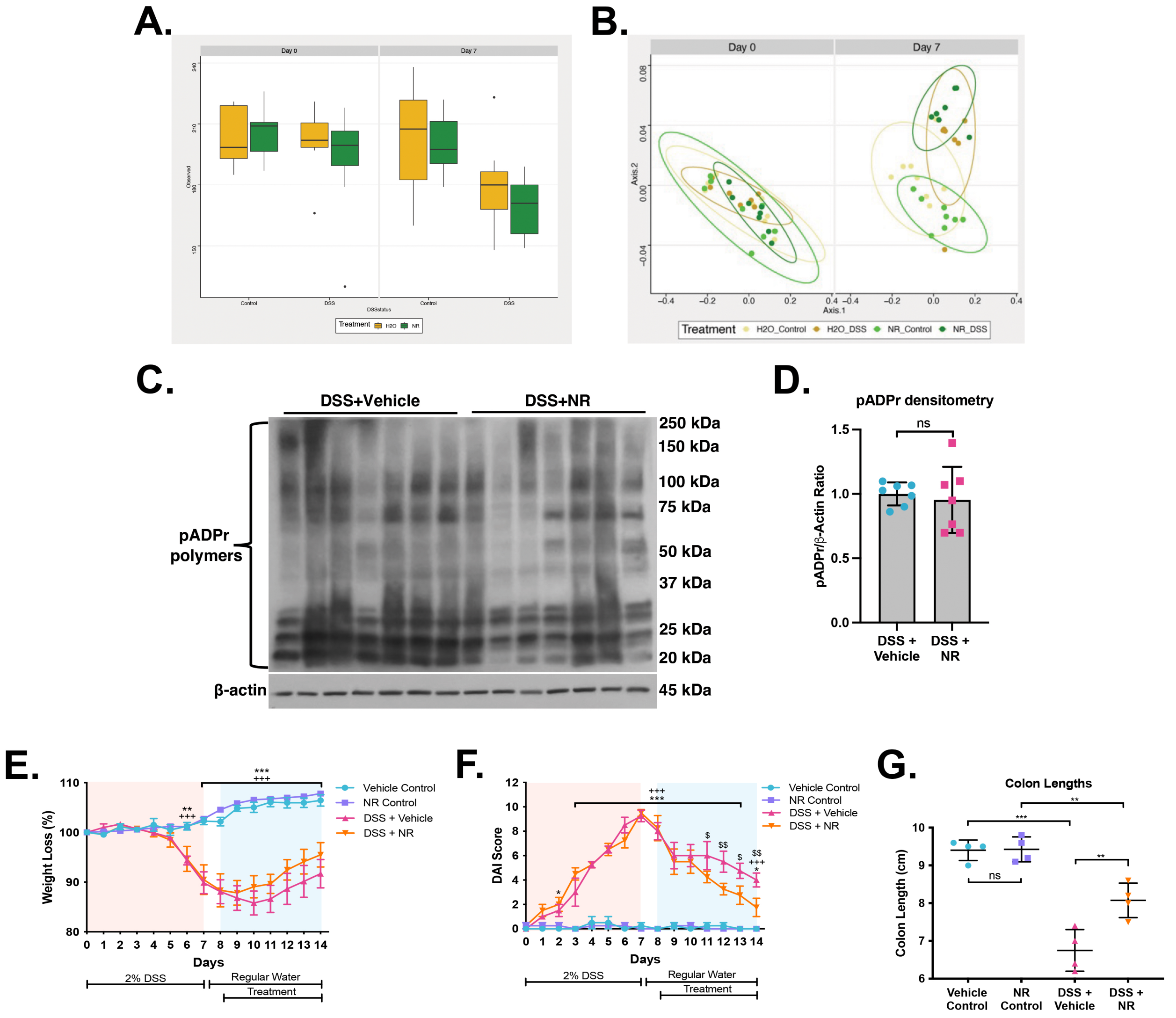
A.
B.
Day 0
Day 7
Treatment
H2O_Control
H2O_DSS
NR_Control
NR_DSS
Axis.1
Axis.2
C.
DSS+Vehicle
DSS+NR
pADPr polymers
β-actin
250 kDa
150 kDa
100 kDa
75 kDa
50 kDa
37 kDa
25 kDa
20 kDa
45 kDa
D.
pADPr densitometry
pADPr/β-Actin Ratio
ns
DSS + Vehicle
DSS + NR
E.
Weight Loss (%)
Days
Vehicle Control
NR Control
DSS + Vehicle
DSS + NR
2% DSS
Regular Water
Treatment
F.
DAI Score
Days
Vehicle Control
NR Control
DSS + Vehicle
DSS + NR
2% DSS
Regular Water
Treatment
G.
Colon Lengths
Colon Length (cm)
ns
Vehicle Control
NR Control
DSS + Vehicle
DSS + NR

Figure S1. NAD$^+$ repletion improved disease in mice subjected to DSS. Acute colitis was induced by giving mice 2% DSS in their water for 7 days. Mice were treated with nicotinamide riboside (NR; 500 mg/kg/day) or vehicle (sterilized water; same mg/kg volume as for NR) once a day during DSS exposure. Stool was collected on Days 0 and 7. **A–B)** Microbiome differences were measured via 16S rRNA sequencing for all groups. **C–D)** Western blot analysis of poly(ADP)-ribose (pADPr)-modified proteins within the intestinal epithelium of DSS+Vehicle mice as compared to DSS+NR mice (n=7/group). **E–G)** NR was administered therapeutically to mice undergoing DSS colitis. Acute colitis was induced by giving mice 2% DSS in their water for 7 days (red area of graph), followed by regular water for 7 days. Mice were treated with NR (500 mg/kg/day) or vehicle (sterilized water; same mg/kg volume as for NR) once a day from Days 8–14 (blue area of graph). **E)** The percent weight loss of each mouse was calculated and averaged as a group. **F)** DAI scores were calculated for each mouse daily and averaged as a group. For E and F, the data are shown as the mean ± SEM; n=4/group. Asterisks (*) indicate a significance difference between DSS+Vehicle vs. DSS+NR groups via a two-way ANOVA. Plus signs (+) indicate a significant difference between Vehicle Control vs. DSS+Vehicle groups via a two-way ANOVA. Dollar signs ($) indicate a significance difference between NR Control vs. DSS+NR groups via a two-way ANOVA. **G)** Colon lengths were measured (n=4/group). Unless indicated otherwise, the data are shown as the mean ± SD. The data are representative of at least two independent experiments. Significance differences between groups [as determined by an unpaired two-tailed *t* test (D), one-way ANOVA (G), or two-way ANOVA (E, F)] are indicated on the graphs. * $p < 0.05$, ** $p < 0.005$, and *** $p < 0.0005$; ns, not significant.

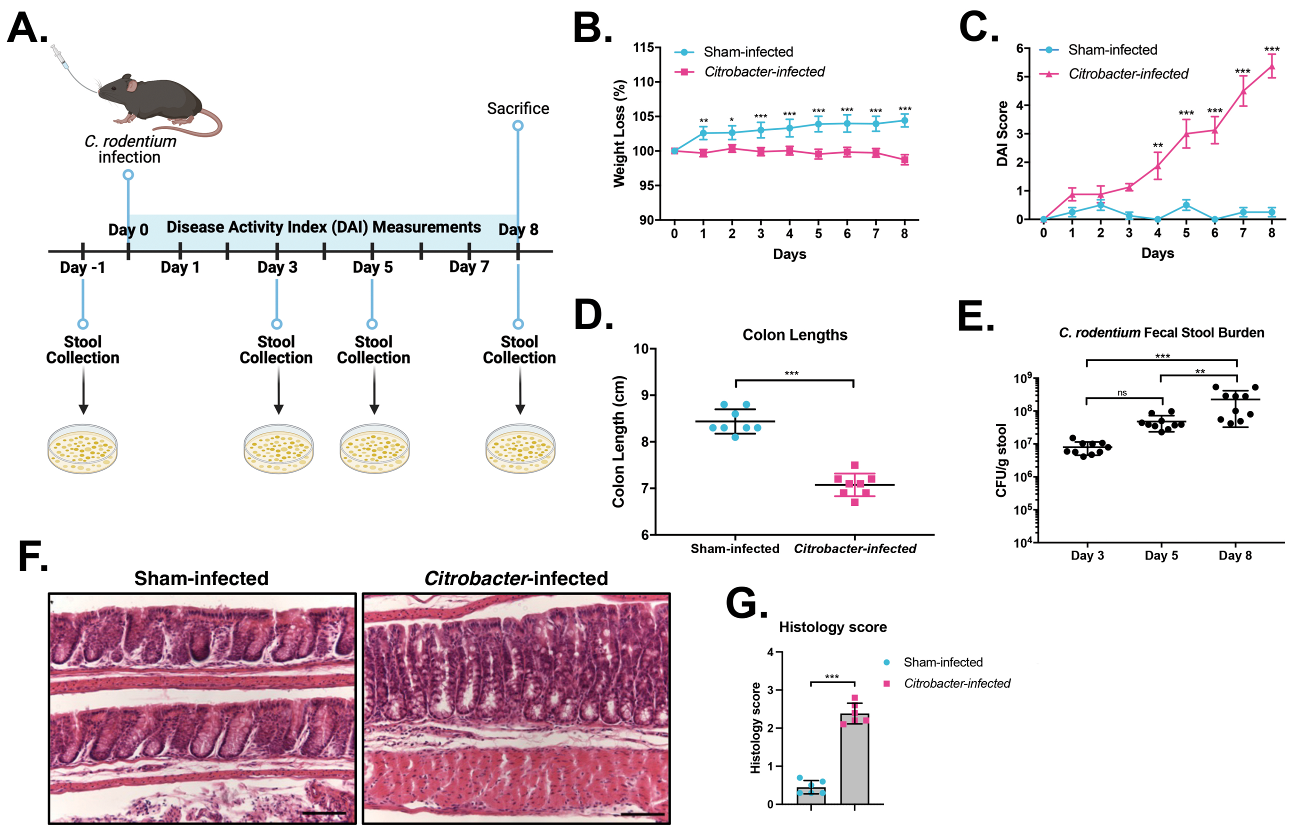
A.
C. rodentium infection
Sacrifice
Day 0
Disease Activity Index (DAI) Measurements
Day 8
Day -1
Day 1
Day 3
Day 5
Day 7
Stool Collection
Stool Collection
Stool Collection
Stool Collection
B.
Sham-infected
Citrobacter-infected
Weight Loss (%)
Days
C.
Sham-infected
Citrobacter-infected
DAI Score
Days
D.
Colon Lengths
Colon Length (cm)
Sham-infected
Citrobacter-infected
E.
C. rodentium Fecal Stool Burden
CFU/g stool
Day 3
Day 5
Day 8
ns
F.
Sham-infected
Citrobacter-infected
G.
Histology score
Histology score
Sham-infected
Citrobacter-infected

Figure S2. Establishment of the infectious colitis model. Infectious colitis was induced by infecting mice with 1×10^9 colony-forming units (CFUs) of *Citrobacter rodentium.* **A)** A schematic outlining the infectious colitis model used in this study. Image was created with BioRender.com. **B)** The percent weight loss of each mouse was calculated and averaged as a group (n=8/group). Data are shown as the mean ± SEM. **C)** Disease activity index (DAI) scores were calculated for each mouse daily and averaged as a group (n=8/group). Data are shown as the mean ± SEM. **D)** Colon lengths were measured (n=8/group). **E)** The fecal stool burden (CFU/g of stool) of *C. rodentium* was determined by plating (n=10/group). **F–G)** Colonic sections were stained via H&E and scored in a blinded manner by a pathologist (n=6/group); scale bar = 100 µM. Unless indicated otherwise, the data are shown as the mean ± SD. The data are representative of at least two independent experiments. Significance differences between groups [as determined by an unpaired two-tailed *t* test (D, G), one-way ANOVA (E), and two-way ANOVA (B, C)] are indicated on the graphs. * $p < 0.05$, ** $p < 0.005$, and *** $p < 0.0005$; ns, not significant.

Supplementary Table

Table S1. qPCR primer sequences used in this study.

Primer Name	Gene	Species	Forward sequence (5'-3')	Reverse Sequence (5'-3')
Rplo	*Rplp0*	Mouse	GGCGACCTGGAAGTCCAACT	CCATCAGCACCACAGCCTTC
Pgc1α	*Ppargc1a*	Mouse	TGGATGAAGACGGATTGCCC	TAGAGACGGCTCTTCTGCCT
Tfam	*Tfam*	Mouse	TCTTGGGAAGAGCAGATGGCTGAA	TCCCAATGACAACTCCGTCTTCCA
Sirt1	*Sirt1*	Mouse	TGACCGATGGACTCCTCACT	ATTGTTCGAGGATCGGTGCC
Parp1	*Parp1*	Mouse	AAGGCGGAGAAGACATTGGG	ACCATCTTCTTGGACAGGCG
Parp2	*Parp2*	Mouse	CACAGCTTGGTGACTTGTTCT	ACTCAGGCTTCAAAGTTTCCTC
Nampt	*Nampt*	Mouse	TGGGGTGAAGACCTGAGACA	TGGCAGCAACTTGTAGCCTT
Nmnat1	*Nmnat1*	Mouse	CCACCACCGAATCATCATGG	AGACTTTCCCACGTATCCACT
Nmnat3	*Nmnat3*	Mouse	TCACCCGTCAATGACAGCTAT	CACCCGAATCCAGTCAGATGT
Naprt1	*Naprt1*	Mouse	TGCTCACCGACCTCTATCAGG	GCGAAGGAGCCTCCGAAAG
Pnp	*Pnp*	Mouse	AAGTTCTGGATGCCGGGAAA	AGAGAGCTACTTGGACCCCA
Nadsyn1	*Nadsyn1*	Mouse	CCAAAGGCAAAGGTGCAAGA	ATGTCCTGAGTGACCGGAGA
Nmrk2	*Nmrk2*	Mouse	CAGGGCCTGAGATGTTCTGA	CATAGGGACCATACAGGACGC
Cd38	*Cd38*	Mouse	TCTCTAGGAAAGCCCAGATCG	AGAAAAGTGCTTCGTGGTAGG
Sirt3	*Sirt3*	Mouse	CTGACTGGTCACGTAGCCTC	CCACACAGAGGGATATGGGC
Sirt4	*Sirt4*	Mouse	TCGAGGGGACAAGGAGGATT	CCAAGAAGAGCTCAGGACCC
Sirt5	*Sirt5*	Mouse	ACTTCTTAACCGCCCTGTGG	TTGGGGCTTGAAGGGTGTTT
Sirt6	*Sirt6*	Mouse	GCCCAACAGCCCTATACTCC	GTGGTTCCTTCAAGTTCCCCT
Il1β	*Il1b*	Mouse	AGTGTGGATCCCAAGCAATACCCA	TGTCCTGACCACTGTTGTTTCCCA
inos	*Nos2*	Mouse	CTGCTGGTGGTGACAAGCACATTT	ATGTCATGAGCAAAGGCGCAGAAC
Tnfα	*Tnf*	Mouse	TTCCGAATTCACTGGAGCCTCGAA	TGCACCTCAGGGAAGAATCTGGAA
RPLO	*RPLP0*	Human	GGCGACCTGGAAGTCCAACT	CCATCAGCACCACAGCCTTC
PGC1α	*PPARGC1A*	Human	TCATGGGTGTCAGCTTTGCT	TGAAATGGTTTGCCCTTGCG
SIRT1	*SIRT1*	Human	TCCAAGGCCACGGATAGGT	GTGGAGGTATTGTTTCCGGC
PARP1	*PARP1*	Human	TGGTCTCCAAGAGTGCCAAC	CCCAAACCTTTGACACTGTGC
